# Estrogen receptor α/HDAC/NFAT axis for delphinidin effects on proliferation and differentiation of T lymphocytes from patients with cardiovascular risks

**DOI:** 10.1038/s41598-017-09933-4

**Published:** 2017-08-24

**Authors:** Ousama Dayoub, Soazig Le Lay, Raffaella Soleti, Nicolas Clere, Gregory Hilairet, Séverine Dubois, Frédéric Gagnadoux, Jérôme Boursier, Maria Carmen Martínez, Ramaroson Andriantsitohaina

**Affiliations:** 1 INSERM UMR1063, UNIV Angers, Université Bretagne Loire, Angers, France; 2 Service d’Endocrinologie Diabétologie Nutrition, Centre hospitalier universitaire, Angers, France; 3 Département de Pneumologie, Centre hospitalier universitaire, Angers, France; 4 Service d’Hépato-gastroentérologie, Centre hospitalier universitaire, Angers, France; 5 Laboratoire HIFIH UPRES EA3859, UNIV Angers, Université Bretagne Loire, Angers, France

## Abstract

Delphinidin, an anthocyanin present in red wine, has been reported to preserve the integrity of endothelium *via* an estrogen receptor alpha (ERα)-dependent mechanism. However, the effect of delphinidin on the immune response in obesity-related inflammation remains unknown. Given the important role of T lymphocytes in obesity-related inflammation, we investigated the effect of delphinidin on proliferation and differentiation of T lymphocytes from healthy subjects and metabolic syndrome patients. Delphinidin decreased the proliferation stimulated by different agents acting through different mechanisms. This effect of delphinidin was associated with its ability to inhibit Ca^2+^ signaling via reduced store-operated Ca^2+^ entry and release, and subsequent decrease of HDAC and NFAT activations. Delphinidin also inhibited ERK1/2 activation. Pharmacological inhibition of ER with fulvestrant, or deletion of ERα, prevented the effect of delphinidin. Further, delphinidin suppressed the differentiation of T cells toward Th1, Th17 and Treg without affecting Th2 subsets. Interestingly, delphinidin inhibited both proliferation and differentiation of T cells taken from patients with cardiovascular risks associated with metabolic syndrome. Together, we propose that delphinidin, by acting on ERα via multiple cellular targets, may represent a new approach against chronic inflammation associated with T lymphocyte activation, proliferation and differentiation, in patients with cardiovascular risk factors.

## Introduction

Obesity and its metabolic complications including metabolic syndrome (MetS) share a common pathogenic denominator of chronic, low-grade inflammation^1, 2^. These disorders are characterized by a continuous activation of several immune cells including T lymphocytes^1^. Activated T lymphocytes play a fundamental role in initiation and development of chronic inflammatory responses. Indeed, increased numbers of T lymphocytes and a modification in the balance between pro-inflammatory (Th1 and Th17 lymphocytes) and anti-inflammatory (Th2 and Treg lymphocytes) CD4^+^ cells subsets have been observed in adipose tissue of obese and MetS patients^3, 4^. Moreover, suppressing of T cell activation protects against obesity and adipose tissue inflammation^5^.

Activation of T lymphocytes through T-cell receptor (TCR) phosphorylates and promotes the translocation of extracellular signal-regulated kinase 1/2 (ERK1/2) into the nucleus to regulate the transcription of target genes^6^. In addition, TCR activation induces an increase in intracellular Ca^2+^ concentration ([Ca^2+^]_i_). The increase of [Ca^2+^]_i_ includes the release of Ca^2+^ from the endoplasmic reticulum via the inositol 1, 4, 5-triphosphate (IP_3_) production and the store-operated Ca^2+^ entry (SOCE) through Ca^2+^ release-activated Ca^2+^ (CRAC) channels^7^. The increase of [Ca^2+^]_i_ triggers different pathways including nuclear factor of activated T-cells (NFAT) and histone deacetylase (HDAC)^8^. These pathways mediate the functional modifications of T lymphocytes observed during chronic inflammation, including T cell proliferation and differentiation toward the pro-inflammatory cells over anti-inflammatory cells^8–10^.

Natural dietary polyphenolic compounds have been reported to protect against cardiovascular diseases and inflammation due to their multitude biological activities^11, 12^. We have previously identified the anthocyanin, delphinidin, as a polyphenol with endothelium-dependent relaxation property by promoting nitric oxide (NO) production^13^. NO production induced by delphinidin involves activation of the isoform alpha of estrogen receptor (ERα)^14^ and increase of [Ca^2+^]_i_^15^. In addition, delphinidin inhibits endothelial cell proliferation by modulating the activity of MAP kinase^16, 17^.

Given the crucial role of T lymphocytes in chronic inflammatory metabolic diseases, modulation of T lymphocytes function by using polyphenols as a possible approach might be of importance^18, 19^. *Jara et al*.^20^ have shown that delphinidin can activate NFAT and induce cytokine production through SOCE in T cells. In contrast, delphinidin suppresses NF-κB acetylation and inhibits cytokine production in Jurkat cells^21^. However, the mechanism by which delphinidin affects T lymphocyte functions is not elucidated yet. Thus, the present study was carried out to analyze the effects of delphinidin on proliferation and differentiation of T lymphocytes isolated from healthy subjects and MetS patients. Further, we have examined the related signaling pathways involved in delphinidin effects.

## Results

### Delphinidin reduces the proliferation of T lymphocytes from healthy subjects

**Figure 1:**
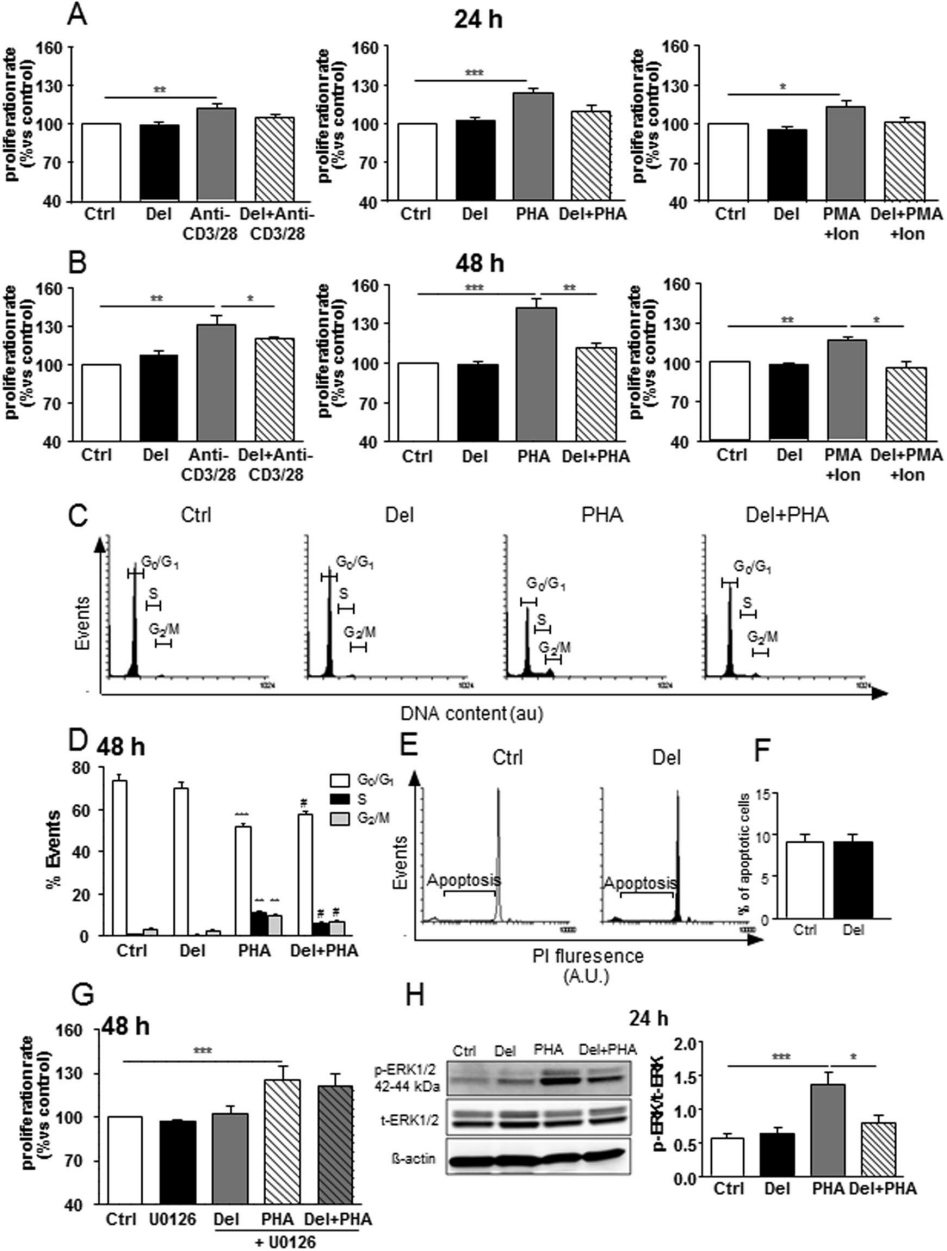
Effects of delphinidin on proliferation, cell cycle progression and ERK1/2 pathway activation of T lymphocytes from healthy subjects. Histograms show the percentage of proliferation of cells exposed to T cell activators [10 µg/mL anti-CD3 plus 5 µg/mL anti-CD28, 5 µg/mL PHA or 10 ng/mL PMA plus 1 µM ionomycin (Ion)] in absence or in presence of 10^−2^ g/L of delphinidin (Del) for 24 h (**A**) or 48 h (**B**). Data are the mean ± SEM (*n* = 5–10). *P < 0.05, **P < 0.01 and ***P < 0.001. (**C**) Representative cytometric profiles showing cells in the G_0_/G_1_, S and G_2_/M phases of the cell cycle after 48 h of treatment with either 10^−2^ g/L of Del, 5 µg/mL PHA or both. (**D**) Histograms show the percentage of the cells in the G_0_/G_1_, S and G_2_/M phases determined by flow cytometric analysis. Data are the mean ± SEM (*n* = 12). **P < 0.01 and ***P < 0.001 versus control group. ^#^ *P* < 0.05 versus PHA group. (**E**) Representative histograms of flow cytometry showing sub-G1 peak, corresponding to the apoptotic population in propidium iodide (PI)-stained cells. (**F**) Quantification of sub-G1 peak. (**G**) Histograms show the percentage of proliferation of cells exposed to either 10^−2^ g/L of delphinidin (Del), 5 µg/mL PHA or both in presence or in absence of 10 µM of specific inhibitor of ERK1/2 pathway (U0126) for 48 h. (**H**) Western blot of phosphorylated ERK1/2 in cells exposed to either 10^−2^ g/L of delphinidin (Del), 5 µg/mL PHA or both during 24 h. Histograms show densitometric analysis of phosphorylated ERK1/2 normalized to total ERK1/2 expression. Data represent the mean ± SEM (*n* = 4–8). *P < 0.05 and ***P < 0.001.

Delphinidin had no effect on basal proliferation of T cells treated for 24 or 48 h (Fig. 1A and B). Anti-CD3 plus anti-CD28 antibodies, PHA or PMA plus ionomycin significantly increased T cell proliferation after 24 and 48 h of treatment. Delphinidin prevented anti-CD3 plus anti-C28-, PHA- and PMA plus ionomycin-induced proliferation of T lymphocytes at 48 h but not at 24 h (Fig. 1A and B). Thus, delphinidin has antiproliferative properties on T lymphocytes isolated from healthy subjects independent of the activated pathway. For the following experiments, the activator PHA has been chosen to study the mechanism by which delphinidin reduces T lymphocyte proliferation.

### Delphinidin impairs cell cycle progression by inducing arrest in G_0_/G_1_ phase

The effect of delphinidin on cell cycle was evaluated at 48 h. Delphinidin had no effect on cell cycle compared to control cells. PHA alone induced a significant decrease of the cell population in G_0_/G_1_ phase, whereas the proportion of cells in S and G_2_/M phases was increased in comparison to control cells (Fig. 1C and D). When T cells were treated with PHA and delphinidin, an increase of cells in G_0_/G_1_ phase, concomitant to a decrease of cells in S and G_2_/M phases was observed when compared to PHA-treated T cells (Fig. 1C and D). Importantly, delphinidin had no effect on T cell apoptosis (Fig. 1E and F). Thus, the antiproliferative effect of delphinidin is associated with suppression of cell progression by blocking the cell cycle in G_0_/G_1_ phase.

### Delphinidin inhibits T lymphocyte proliferation through ERK1/2 kinase-dependent pathway

The potential implication of ERK1/2 in the antiproliferative effect of delphinidin was tested using a specific ERK1/2 pathway inhibitor (U0126)^6, 22–24^. U0126 alone did not affect the proliferation of T cells isolated from healthy subjects (Fig. 1G). Noticeably, in the presence of U0126, delphinidin failed to reduce proliferation induced by PHA (Fig. 1G).

To confirm these results, ERK1/2 activation was assessed by Western blot after 10, 30 min, 1, 24 and 48 h of T cells treated with PHA, delphinidin or both. As shown in Supplementary Fig. 1, an increase of ERK1/2 phosphorylation was observed at 10 min and maintained even after 30 min, or 24 h following PHA stimulation. Delphinidin had no effect on basal ERK1/2 activity at any time points, but it prevented the PHA-induced ERK1/2 activation after 24 h of PHA stimulation (Fig. 1H). Altogether, these results suggest that delphinidin inhibits T lymphocyte proliferation through ERK1/2 kinase-dependent pathway. Moreover, they support the hypothesis that ERK1/2 occurred at the early event leading to cell proliferation.

### Delphinidin inhibits T lymphocyte proliferation through SOCE-dependent pathway

**Figure 2:**
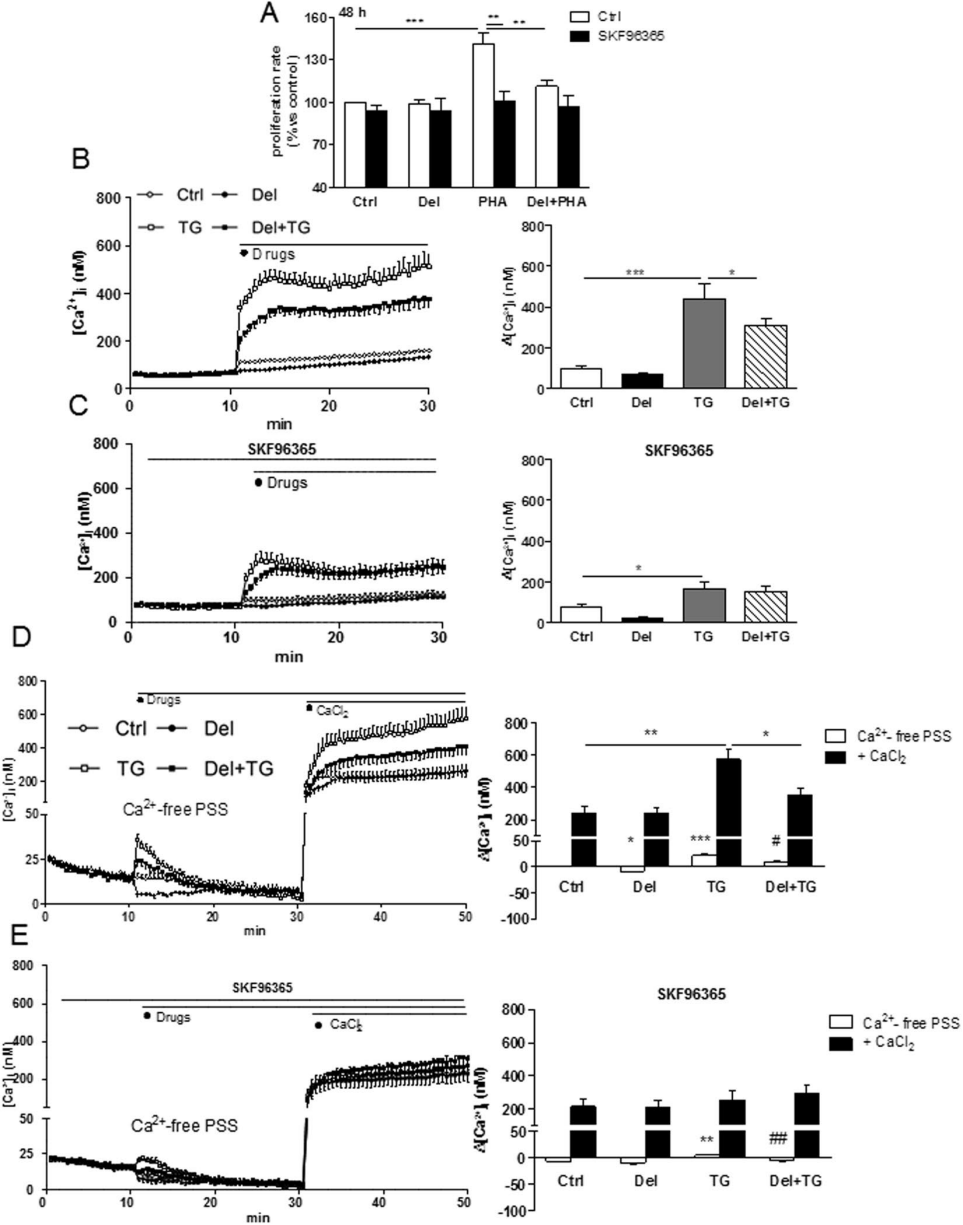
Delphinidin inhibits T lymphocyte proliferation through SOCE-dependent pathway. (**A**) Histograms show the percentage of proliferation of cells exposed to either 10^−2^ g/L of delphinidin (Del), 5 µg/mL PHA or both, in absence or in presence of 10 µM of SOCE inhibitor (SKF96365) for 48 h. (**B**) Representative traces (*left*) and histograms (*right*) showing the effect of 10^−2^ g/L of delphinidin (Del) alone or after activation by 1 µM thapsigargin (TG) on [Ca^2+^]_i_ in Ca^2+^-containing PSS. (**C**) Same experiments in presence of 10 µM of SOCE inhibitor (SKF96365). (**D**) Representative traces (*left*) and histograms (*right*) showing the effect of 10^−2^ g/L delphinidin on [Ca^2+^]_i_ increase induced by 1.25 mM of CaCl_2_ after depletion of intracellular stores in Ca^2+^-free PSS by 1 µM TG. (**E**) Same experiments in presence of 10 µM of SOCE inhibitor, SKF96365. Data are the mean ± SEM (*n* = 7–11). *P < 0.05, **P < 0.01 and ***P < 0.001 *versus* control group. ^#^ *P* < 0.05 and ^##^ *P* < 0.01 *versus* TG group.

T lymphocyte activation induces an augmentation of [Ca^2+^]_i_ principally by SOCE, which is an essential step in their proliferation^25, 26^. The SOCE inhibitor, SKF96365, had no effect on T cell proliferation, but it prevented the PHA-induced proliferation after 48 h of treatment (Fig. 2A). Delphinidin failed to reduce further T cell proliferation in presence of PHA plus SKF96365 (Fig. 2A). Then, the effect of delphinidin was investigated on Ca^2+^ signaling. Delphinidin had no effect on basal [Ca^2+^]_i_ but it significantly decreased the thapsigargin-induced [Ca^2+^]_i_ increase in Ca^2+^-containing PSS (Fig. 2B). SKF96365 reduced the increase of [Ca^2+^]_i_ induced by thapsigargin. Interestingly, the effect of delphinidin on the thapsigargin-induced [Ca^2+^]_i_ increase was completely abrogated in presence of SKF96365 (Fig. 2C).

In order to investigate the source of Ca^2+^ implicated in the activation of T lymphocytes, the effects of delphinidin on [Ca^2+^]_i_ increase induced by CaCl_2_ addition after depletion of intracellular stores by thapsigargin in Ca^2+^-free solution was investigated. Delphinidin reduced basal [Ca^2+^]_i_ in Ca^2+^-free PSS. Moreover, delphinidin reduced both the thapsigargin-induced [Ca^2+^]_i_ increase resulting from the release of Ca^2+^ from intracellular stores and the Ca^2+^ influx from extracellular medium after CaCl_2_ addition (Fig. 2D). The effect of delphinidin on thapsigargin-induced [Ca^2+^]_i_ increase resulting from the influx of extracellular Ca^2+^ was completely abolished in presence of SKF96365 (Fig. 2E). Altogether, these data highlight that the antiproliferative effect of delphinidin is associated with its ability to inhibit Ca^2+^ influx through SOCE.

### Delphinidin inhibits T lymphocyte proliferation through NFAT-dependent pathway

**Figure 3:**
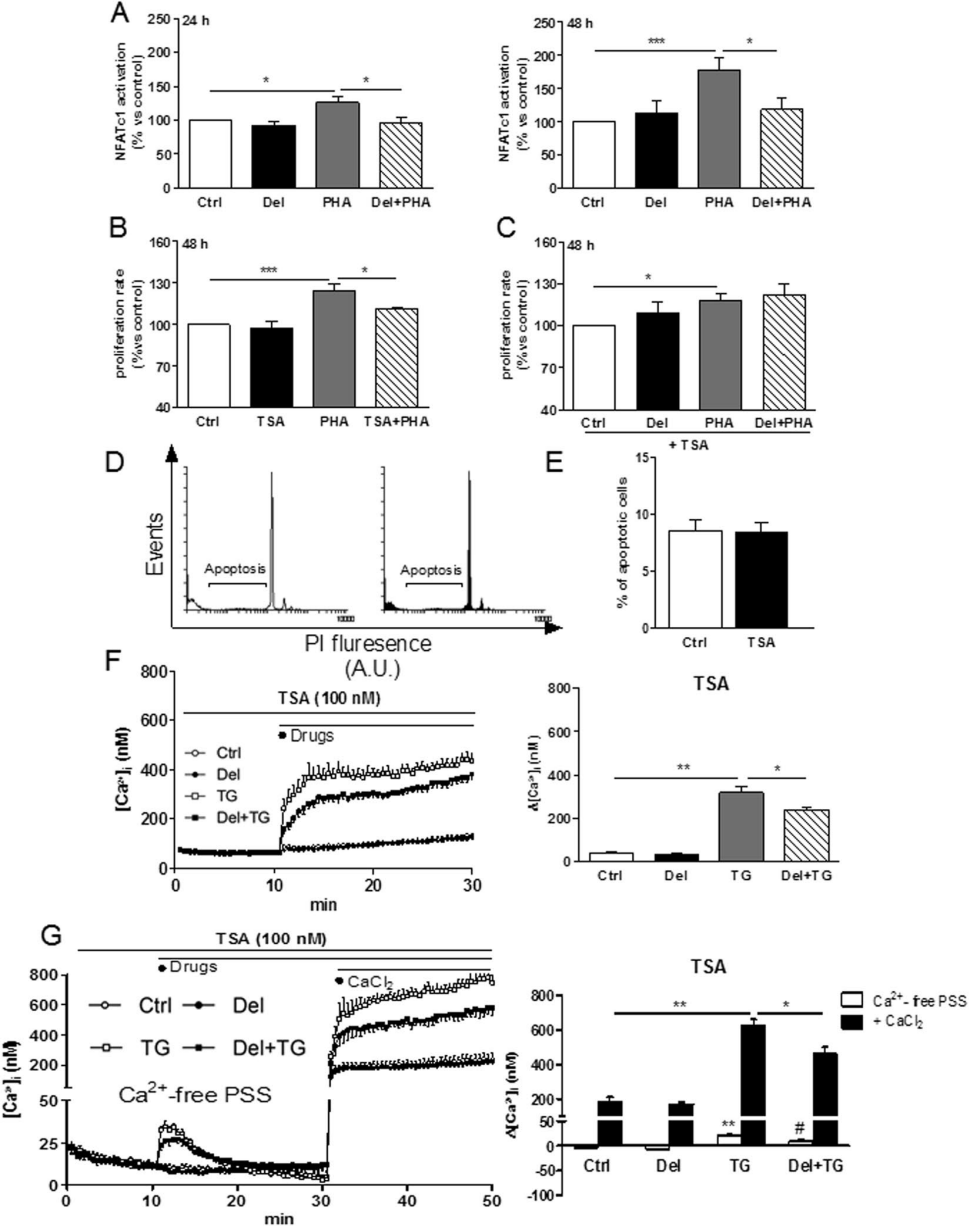
Delphinidin inhibits T lymphocyte proliferation through NFAT and HDAC-dependent pathway. (**A**) Histograms show the percentage of NFAT activation of cells exposed to either 10^−2^ g/L of delphinidin (Del), 5 µg/mL PHA or both for 24 and 48 h. (**B**) Histograms show the percentage of proliferation of cells exposed to HDAC inhibitor (TSA, 100 nM), 5 µg/mL PHA or both for 48 h. (**C**) Histograms show the percentage of proliferation of cells exposed to either 10^−2^ g/L of delphinidin (Del), 5 µg/mL PHA or both in presence of 100 nM of TSA for 48 h. Data are the mean ± SEM (*n* = 5). *P < 0.05 and ***P < 0.001. (**D**) Representative histograms of flow cytometry showing sub-G1 peak, corresponding to the apoptotic population in propidium iodide (PI)-stained cells. (**E**) Quantification of sub-G1 peak. (**F**) Representative traces (*left*) and histograms (*right*) showing the effect of 10^−2^ g/L of delphinidin (Del) alone or after activation by 1 µM thapsigargin (TG) on [Ca^2+^]_i_ in Ca^2+^-containing PSS in presence of 100 nM of TSA. (**G**) Representative traces (*left*) and histograms (*right*) showing the effect of 10^−2^ g/L delphinidin on [Ca^2+^]_i_ increase induced by 1.25 mM of CaCl_2_ after depletion of intracellular stores in Ca^2+^-free PSS by 1 µM TG in presence of 100 nM of TSA. Data are the mean ± SEM (*n* = 5–8). *P < 0.05 and **P < 0.01. **P < 0.01 *versus* control group, ^#^ *P* < 0.05 *versus* TG group.

The implication of NFAT pathway in the antiproliferative effect of delphinidin was investigated. Delphinidin alone had no effect on NFAT activation compared to control cells (Fig. 3A). Interestingly, delphinidin decreased the PHA-induced NFAT activation at 24 and 48 h demonstrating that the antiproliferative effect of delphinidin is associated with its ability to inhibit NFAT activation (Fig. 3A).

### Delphinidin inhibits T lymphocyte proliferation through HDAC-dependent pathway

The role of HDAC in the antiproliferative properties of delphinidin was investigated using the HDAC inhibitor (TSA). TSA did not modify the basal proliferation of T cells but it was able to inhibit the PHA-induced proliferation (Fig. 3B). Furthermore, at 48 h, the antiproliferative effect of delphinidin was completely prevented in presence of TSA (Fig. 3C). The concentration of TSA used in this study did not induce apoptosis (Fig. 3D and E). Thus, delphinidin prevents the proliferation of T lymphocytes by a mechanism sensitive to inhibition of HDAC activity.

The role of Ca^2+^ signaling in the HDAC-dependent antiproliferative effect of delphinidin was analyzed. TSA had no effect on the basal [Ca^2+^]_i_ nor on the thapsigargin-induced [Ca^2+^]_i_ increase in both Ca^2+^-containing and Ca^2+^-free PSS (Supplementary Fig. 2). Interestingly, delphinidin was still able to reduce the ability of thapsigargin to increase [Ca^2+^]_i_ in the presence of TSA (Fig. 3F and G). These results suggest that delphinidin inhibits HDAC activity by a mechanism downstream of its effect on Ca^2+^ signaling.

### Delphinidin inhibits T lymphocyte proliferation through ERα-dependent mechanism

**Figure 4:**
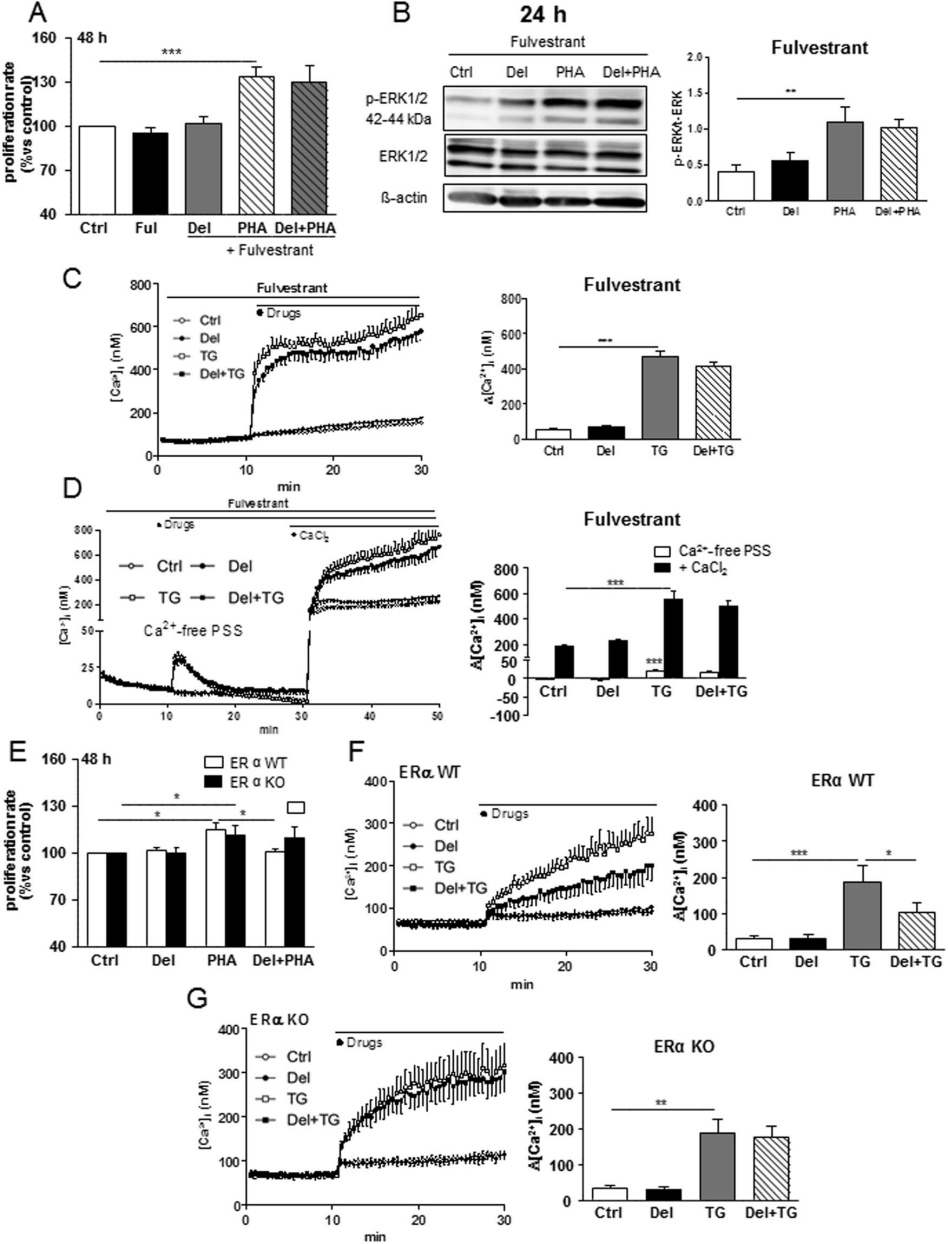
Delphinidin inhibits T lymphocyte proliferation through ERα-dependent mechanism. (**A**) Histograms show the percentage of proliferation of cells exposed to either 10^−2^ g/L of delphinidin (Del), 5 µg/mL PHA or both in presence or in absence of nonselective estrogen receptor antagonist (Fulvestrant 100 nM) for 48 h. Data are the mean ± SEM (*n* = 8). (**B**) Western blot of phosphorylated ERK1/2 in T cells exposed to either 10^−2^ g/L of delphinidin, 5 µg/mL PHA or both for 24 h in presence of fulvestrant. Histograms show densitometric analysis of phosphorylated ERK1/2 normalized to total ERK1/2 expression, Data are the mean ± SEM (*n* = 6). (**C**) Representative traces (*left*) and histograms (*right*) showing the effect of 10^−2^ g/L of delphinidin alone or after activation by 1 µM thapsigargine (TG) on [Ca^2+^]_i_ in Ca^2+^-containing PSS in presence of Ful (100 nM). (**D**) Representative traces (*left*) and histograms (*right*) showing the effect of delphinidin on [Ca^2+^]_i_ increase induced by 1.25 mM of CaCl_2_ after depletion of intracellular stores in Ca^2+^-free PSS by thapsigargin in presence of fulvestrant. Data are the mean ± SEM (*n* = 7). (**E**) Histograms show the percentage of proliferation of cells isolated from ERα WT or KO mice exposed to either 10^−2^ g/L of delphinidin, 5 µg/mL PHA or both for 48 h. (**F**) Representative traces (*left*) and histograms (*right*) showing the effect of 10^−2^ g/L of delphinidin (Del) alone or after activation by 1 µM thapsigargin (TG) on [Ca^2+^]_i_ in Ca^2+^-containing PSS of cells isolated from ERα WT mice. (**G**) Same experiments on cells isolated from ERα KO mice. Data are the mean ± SEM (*n* = 4). *P < 0.05, **P < 0.01 and ***P < 0.001 *versus* control group.

Next, we investigated the implication of ERα in controlling the antiproliferative properties of delphinidin. The ER antagonist, fulvestrant, at a concentration at which it had no effect by itself on T cell proliferation, abolished the antiproliferative effect of delphinidin (Fig. 4A). Interestingly, fulvestrant also prevented the ability of delphinidin to decrease the PHA-induced ERK1/2 phosphorylation (Fig. 4B). Moreover, fulvestrant prevented the ability of delphinidin to inhibit the increase of [Ca^2+^]_i_ induced by thapsigargin both in Ca^2+^-containing and Ca^2+^-free PSS (Fig. 4C and D).

To further characterize the ER isoform involved in delphinidin effects, the same experimental protocols were conducted in peripheral blood mononuclear cells (PBMCs) isolated either from ERα WT or KO mice. Interestingly, delphinidin prevented the PHA-induced proliferation in PBMCs isolated from WT but not from KO mice (Fig. 4E). In addition, delphinidin decreased the thapsigargin-induced [Ca^2+^]_i_ increase in T cells isolated from WT but not from KO mice (Fig. 4F and G). Altogether, these results demonstrate that delphinidin exerts its antiproliferative effect resulting from inhibition of Ca^2+^ entry and ERK1/2 activation via the α isoform of the ER.

### Delphinidin decreases Th1, Th17, Treg but not Th2 differentiation of T lymphocytes from healthy subjects

**Figure 5:**
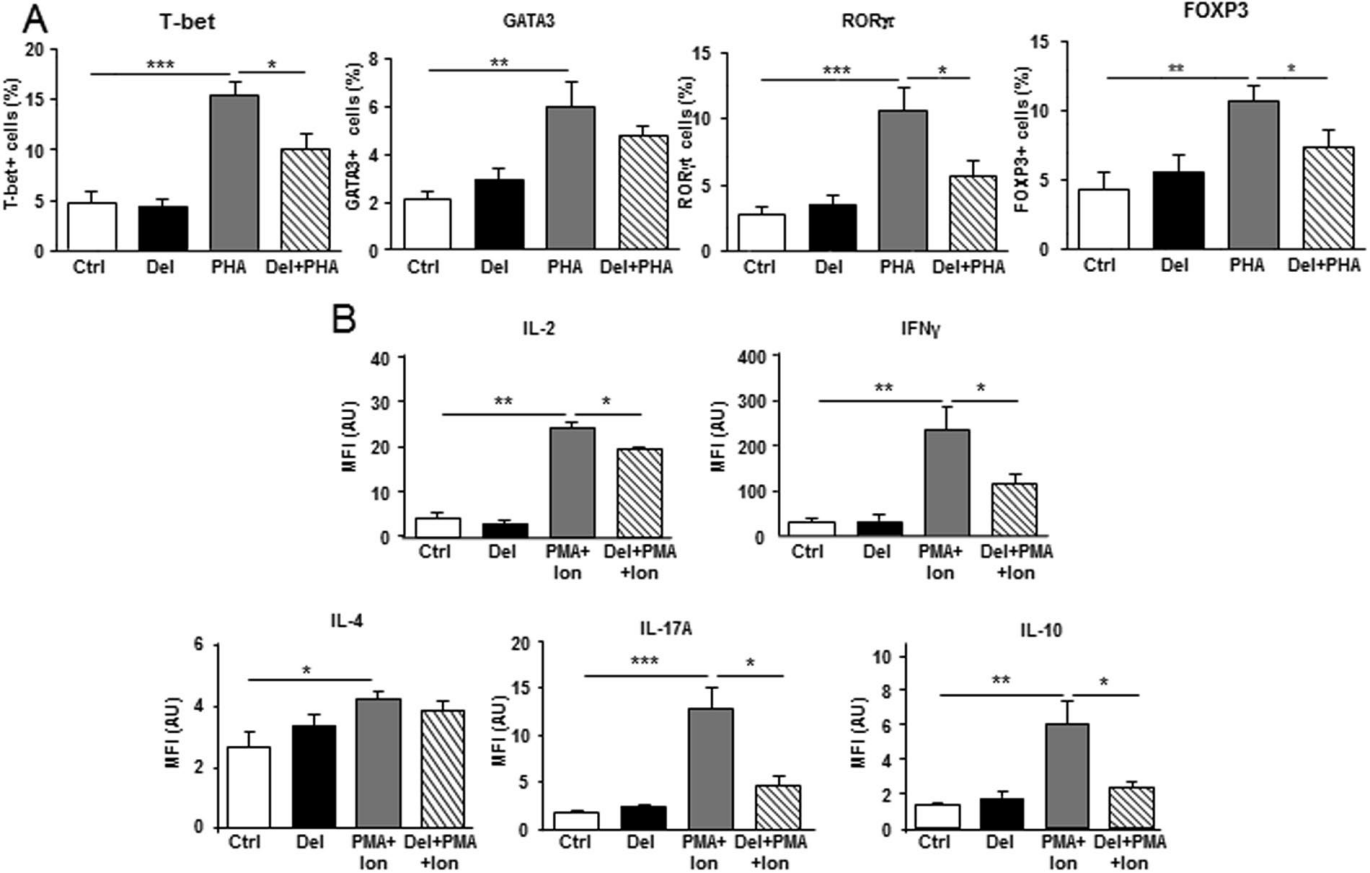
Effect of delphinidin on the differentiation of T lymphocytes from healthy subjects. (**A**) Cells were stimulated for 24 h with 10^−2^ g/L of delphinidin (Del), 5 µg/mL of PHA or both and stained for T-bet, GATA3, RORγt and FOXP3 transcription factors. Histograms show the percentage of positive cells for T-bet, GATA3, RORγt and FOXP3 expressions. Data are the mean ± SEM (*n* = 7). (**B**) T cells were stimulated for 5 h with 10^−2^ g/L of delphinidin (Del), 50 ng/mL phorbol-12-myristate-13-acetate (PMA) plus 1 µg/mL ionomycin (Ion) or both, in the presence of 5 µg/mL brefeldin A (BFA) for the last 3 h of culture and stained for IL-2, IFNγ, IL-4, IL-17A and IL-10 cytokines. Histograms show the MFI of positive cells for IL-2, IFNγ, IL-4, IL-17A, and IL-10, respectively. Data are the mean ± SEM (*n* = 7). *P < 0.05, **P < 0.01 and ***P < 0.001.

The effect of delphinidin on the differentiation of T lymphocytes from healthy subjects following 24 h of PHA treatment was analyzed. As shown in Fig. 5A and Supplementary Fig. 3, PHA increased the expression of T-bet, GATA3, RORγt and FOXP3 (the transcription factors that control Th1, Th2, Th17 and Treg, respectively) compared to control cells. Delphinidin had no effect on basal expression of these factors but it was able to decrease the PHA-induced T-bet, RORγt and FOXP3 but not GATA3 expression.

To confirm these results, we investigated the production of IFNγ, IL-4, IL-17A, IL-10 (produced by Th1, Th2, Th17 and Treg, respectively), and IL-2, following delphinidin treatment in activated T cells. In order to get a high production of cytokines over a short time, T lymphocytes were stimulated by PMA plus ionomycin during 5 h. T cells treated with PMA plus ionomycin displayed an increase in IL-2, IFNγ, IL-4, IL-17A and IL-10 production (Fig. 5B, Supplementary Fig. 4). Delphinidin did not modify basal production of any cytokines, whereas it reduced the increase of IL-2, IFNγ, IL-17A, IL-10 but not IL-4 production induced by PMA plus ionomycin. These results suggest that delphinidin inhibits T cell differentiation toward Th1, Th17, Treg but not Th2 subsets.

### Delphinidin reduces proliferation and Ca^2+^ signaling in T lymphocytes from patients with cardiovascular risk factors displaying one or two criteria of MetS

**Figure 6:**
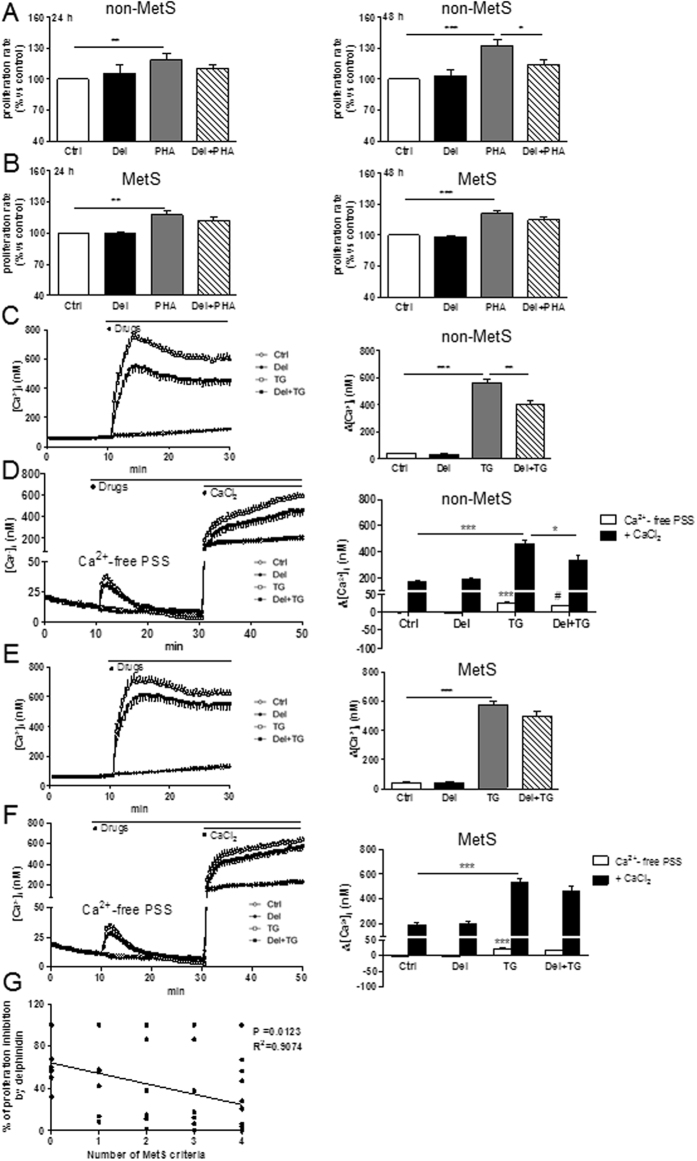
Effects of delphinidin on proliferation and Ca^2+^ signaling of T lymphocytes from non-MetS and MetS patients. (**A**) Histograms show the percentage of proliferation of cells isolated from non-MetS patients exposed to either 10^−2^ g/L of delphinidin (Del), 5 µg/mL PHA or both for 24 and 48 h. (**B**) Same experiments on cells isolated from MetS patients. Data are the mean ± SEM (*n* = 8–31). (**C**) Representative traces (*left*) and histograms (*right*) showing the mean of the responses induced by 10^−2^ g/L of delphinidin (Del) alone or after activation by 1 µM thapsigargin (TG) on [Ca^2+^]_i_ in Ca^2+^-containing PSS of T cells isolated from non-MetS patients. (**D**) Representative traces (*left*) and histogram (*right*) showing the effect of 10^−2^ g/L Del on [Ca^2+^]_i_ increase induced by 1.25 mM of CaCl_2_ after depletion of intracellular stores in Ca^2+^-free PSS by 1 µM TG of cells isolated from non-MetS patients. Data are the mean ± SEM (*n* = 12–14). (**E** and **F**) Same experiments as (**C** and **D**, respectively) on cells isolated from MetS patients. (**G**) Negative correlation between Del effect on PHA-induced proliferation and number of MetS criteria. Data are the mean ± SEM (*n* = 16). *P < 0.05, **P < 0.01 and ***P < 0.001. ***P < 0.001 *versus* control group, ^#^ *P* < 0.05 *versus* TG group.

Immuno-modulatory and anti-inflammatory properties of delphinidin in T lymphocytes from healthy subjects highlighted the potential of this polyphenol to modulate chronic inflammation associated with metabolic diseases. To confirm this hypothesis, the effects of delphinidin on proliferation and Ca^2+^ signaling of T lymphocytes from non-MetS and MetS patients were studied. Delphinidin did not modify the basal proliferation of T lymphocytes from non-MetS and MetS patients. Delphinidin displayed antiproliferative effects on T lymphocytes from non-MetS patients but not those from MetS patients treated for 48 h with PHA (Fig. 6A and B).

Delphinidin did not alter basal [Ca^2+^]_i_ in T cells from non-MetS patients, but it decreased the thapsigargin-induced [Ca^2+^]_i_ increase in Ca^+2^-containing PSS (Fig. 6C). Furthermore, delphinidin reduced the thapsigargin-induced [Ca^2+^]_i_ increase resulting from Ca^2+^ release and Ca^2+^ influx after CaCl_2_ addition (Fig. 6D). Conversely, delphinidin did not alter the basal [Ca^2+^]_i_ nor the thapsigargin-induced [Ca^2+^]_i_ increase (Fig. 6E and F) in T cells from MetS patients.

Altogether, these results highlight the potential of delphinidin to reduce proliferation and Ca^2+^ signaling in T lymphocytes isolated from patients with cardiovascular risk factors displaying one or two criteria of MetS. Accordingly, a negative correlation between the number of MetS criteria and the inhibition of proliferation by delphinidin has been observed (Fig. 6G).

### Delphinidin decreases Th17 and Treg differentiation of T lymphocytes from patients with cardiovascular risk factors displaying one or two criteria of MetS

**Figure 7:**
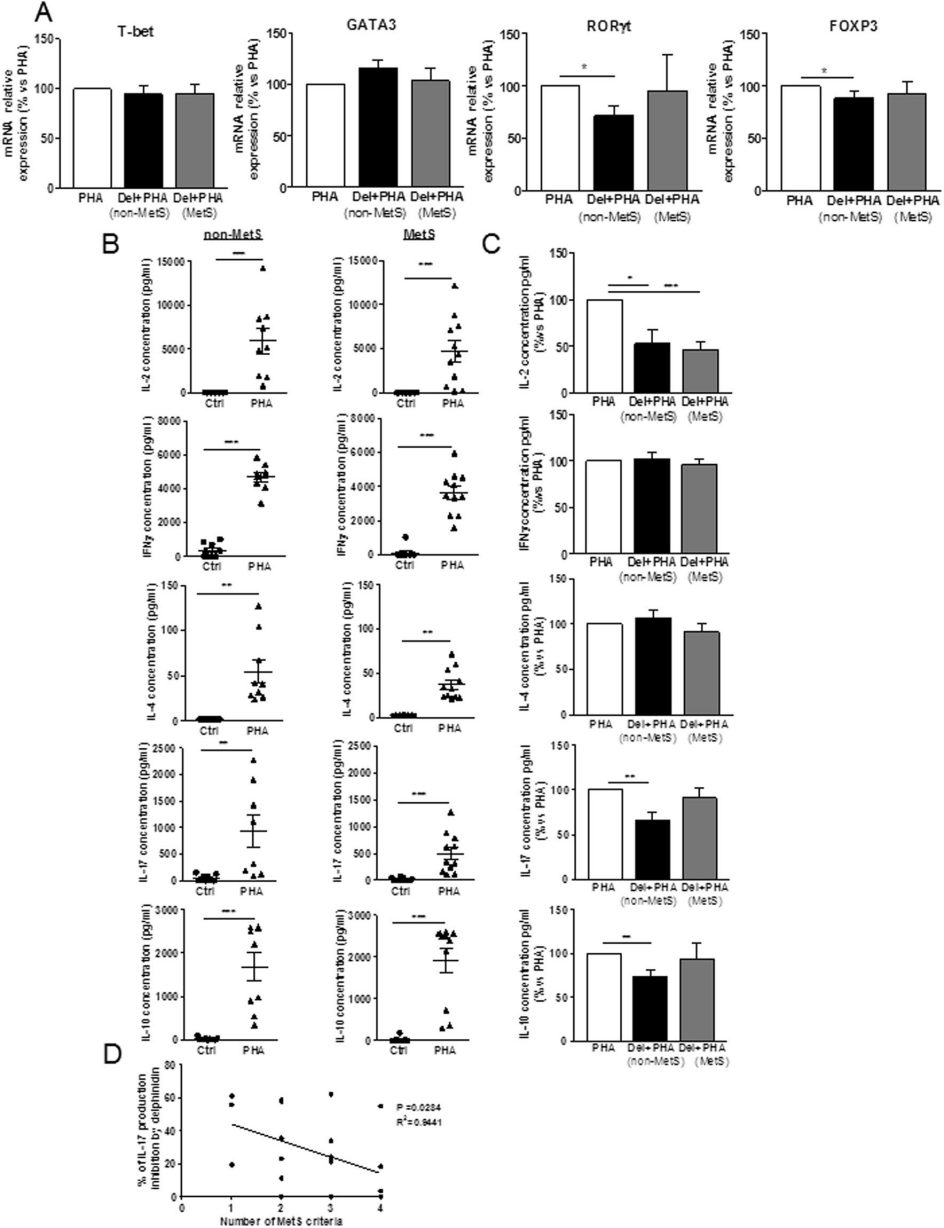
Effect of delphinidin on the differentiation of T lymphocytes from non-MetS and MetS patients. Cells isolated from non-MetS (1–2 criteria) and MetS (3, 4 or 5 criteria) patients were stimulated for 24 h with 10^−2^ g/L of delphinidin (Del), 5 µg/mL of PHA or both. (**A**) The expression of T-bet, GATA3, RORγt and FOXP3 transcription factors in treated cells was measured by RT-qPCR. Results are normalized with the gene reference and expressed as relative expression compared to PHA group. Data are the mean ± SEM (*n* = 5–10). The production of IL-2, IFNγ, IL-4, IL-17 and IL-10 was measured in supernatants of treated cells using Magnetic Luminex Screening Assay. (**B**) Effects of PHA on cytokine production compared to controls cells. (**C**) The effect of delphinidin on cytokines production induced by PHA in non-MetS and MetS-derived cells. Data are the mean ± SEM (*n* = 9–11). (**D**) Negative correlation between delphinidin effect on PHA-induced IL-17 production and number of MetS criteria. *P < 0.05, **P < 0.01 and ***P < 0.001.

The effect of delphinidin on the differentiation of T lymphocytes from non-MetS and MetS patients following 24 h of PHA treatment was further investigated. Delphinidin alone had no effects on T-bet, GATA3, RORγt and FOXP3 expression (data not shown). Alternatively, delphinidin reduced the PHA-induced RORγt and FOXP3 expression in T cells from non-MetS patients, while it had no effects on T cells from MetS patients (Fig. 7A). PHA-induced-T-bet and GATA3 expression of T cells from both non-MetS and MetS patients were unchanged by delphinidin (Fig. 7A).

To further confirm these results, the secretion of IL-2, IFNγ, IL-4, IL-17 and IL-10 in the corresponding cellular supernatants was assessed. PHA increased all cytokines of T cells from non-MetS and MetS patients (Fig. 7B). Delphinidin did not modify basal production of any cytokines (data not shown). Interestingly, it reduced the PHA-stimulated secretion of pro-inflammatory cytokine IL-2, whatever the source of T cells (Fig. 7C). Alternatively, delphinidin decreased IL-17A and IL-10 secretion only in non-MetS patients-derived T cells treated with PHA. PHA-induced IFNγ and IL-4 secretion were unmodified by delphinidin in both T cell types (Fig. 7C). Therefore, modulation of cytokines secretion by T lymphocytes in response to delphinidin was influenced by the number of cardiovascular risk factors, as illustrated by the negative correlation found between the number of MetS criteria and the inhibition of IL-17 secretion by delphinidin (Fig. 7D). Altogether, these results suggest that delphinidin inhibits T cell differentiation toward Th17 and Treg subsets of T lymphocytes from patients with cardiovascular risks displaying one or two criteria of MetS.

## Discussion

**Figure 8:**
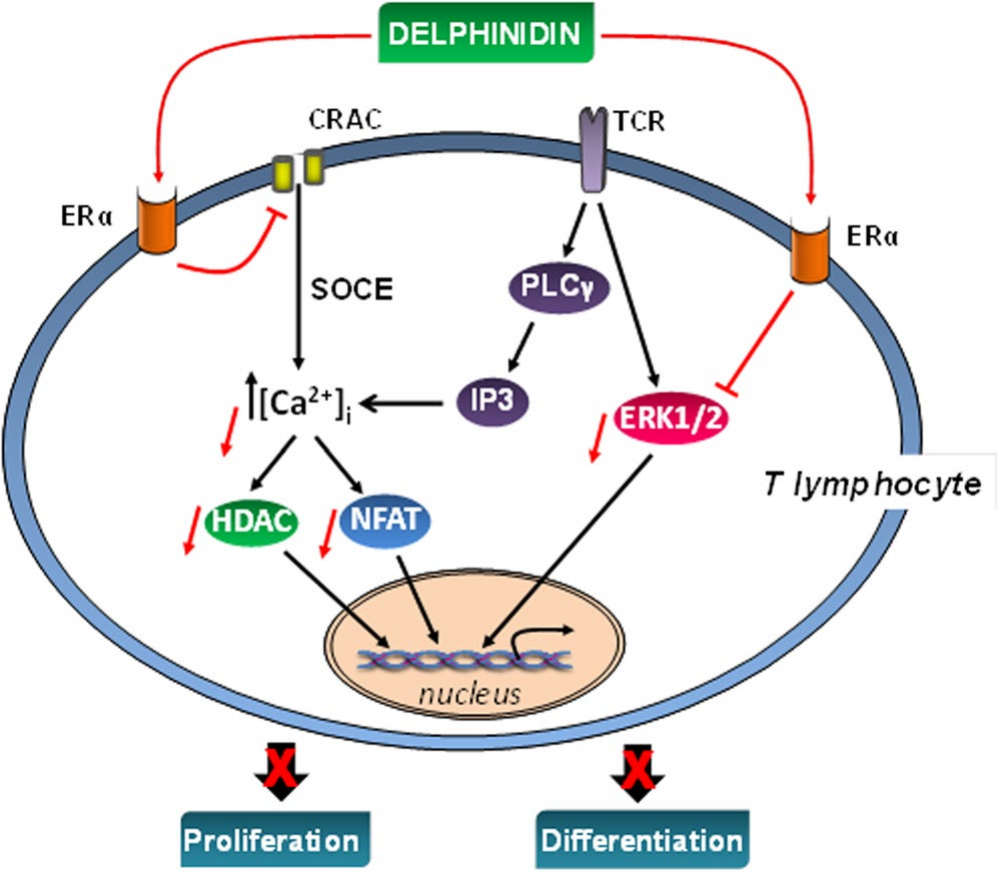
Summary of the mechanisms of the biological effects of delphinidin on T lymphocytes. Delphinidin inhibits ERK1/2, NFAT and HDAC pathways activation, also it decreases [Ca^2+^]_i_ augmentation by inhibiting intracellular Ca^2+^ release and extracellular Ca^2+^ entry through CRAC channels. These effects occur mostly via ERα-dependent mechanism resulting in inhibition of proliferation and differentiation of T lymphocytes.

The present study shows that delphinidin inhibits proliferation of T lymphocytes from healthy subjects and patients with cardiovascular risk factors displaying one or two criteria of MetS by preventing the progression into S and G_2_/M phases of the cell cycle. The antiproliferative effect of delphinidin is related to its capacity to inhibit ERK1/2, NFAT and HDAC pathways. Interestingly, delphinidin decreases [Ca^2+^]_i_ signaling by inhibiting intracellular Ca^2+^ release and extracellular Ca^2+^ entry through CRAC channels. In addition, delphinidin inhibits the differentiation of T lymphocytes from healthy subjects toward Th1, Th17, Treg but not Th2 subsets. The inhibitory effects of delphinidin on Th17 and Treg differentiation are moreover conserved in T cells from patients with cardiovascular risk factors. The present study identifies ERα as the key receptor mediating the immunomodulatory effect of delphinidin (Fig. 8).

Abnormal T lymphocyte activation is one of the major causes of chronic inflammation-associated diseases^27, 28^. Since obesity-related metabolic diseases are associated with an increase in T lymphocyte proliferation^4^, inhibition of T cell proliferation has been reported to have beneficial effects against such diseases^5, 29–32^. We reported that delphinidin inhibited the proliferation of T lymphocytes from healthy subjects stimulated by different agents such as anti-CD3 plus anti-CD28, PHA or PMA plus ionomycin. Thus, delphinidin may have beneficial effects in treating chronic inflammatory metabolic diseases by inhibiting T lymphocyte proliferation.

Cell cycle analysis showed that delphinidin inhibited progression into S and G_2_/M phases of cell cycle of T cells. Consequently, the cells accumulate in G_0_/G_1_ phase. Since delphinidin treatment did not induce apoptosis, the observed accumulation in G_0_/G_1_ phase reflected a specific effect of delphinidin on cell cycle progression rather than a decrease of cell number due to apoptosis. These observations are in agreement with our former data showing that delphinidin impairs cell cycle progression of endothelial cells through blocking G_1_/S transition^17^.

TCR activation triggers several pathway implicated in T cell proliferation, including ERK1/2^6^. Blocking ERK1/2 activation has been identified as one of the mechanisms by which anthocyanins, including delphinidin, exert their antiproliferative activity towards multiple cancer cell types^33–35^. The antiproliferative effect of delphinidin was abolished in presence of ERK1/2 pathway inhibitor, U0126. Furthermore, delphinidin was able to decrease the PHA-induced ERK1/2 activation after 24 h of treatment. Thus, it acts at the early event of T cell proliferation observed at 48 h.

[Ca^2+^]_i_ increase plays an important role in T lymphocyte proliferation^25^. Herein, delphinidin inhibited [Ca^2+^]_i_ increase induced by thapsigargin, used to mimic TCR stimulation via an increase in [Ca^2+^]_i_ resulting from Ca^2+^ release from internal source followed by SOCE. Interestingly, the effect of delphinidin on the thapsigargin-induced [Ca^2+^]_i_ increase was completely abrogated in presence of SKF96365. In addition, the antiproliferative effect of delphinidin was abolished in presence of SKF96365. It should be noted that the pharmacological inhibitor of voltage-dependent Ca^2+^ channel, mibefradil, used at maximally active concentration of 3 µM, did not affect the response to thapsigargin (Supplementary Fig. 5). Moreover, delphinidin was able to reduce Ca^2+^ signaling induced by thapsigargin in the presence of this inhibitor (Supplementary Fig. 5), demonstrating that voltage-operated Ca^2+^ channels were not involved neither in the response to thapsigargin nor the inhibitory effect of delphinidin, under the experimental condition used. Altogether, it can be hypothesized that delphinidin reduces T cell proliferation most likely via inhibition of Ca^2+^ entry via SOCE.

[Ca^2+^]_i_ increase triggers NFAT pathway that controls T lymphocyte proliferation^36, 37^. Delphinidin decreased the PHA-induced NFAT activity after 24 and 48 h. These results suggest that inhibiting TCR-Ca^2+^-NFAT axis is one of the mechanisms by which delphinidin exerts its antiproliferative properties on T cells.

Histone acetylation–deacetylation is an important Ca^2+^-dependent epigenetic event that control T cell proliferation^38, 39^. HDAC inhibitors decrease T cell proliferation without affecting [Ca^2+^]_i_^40, 41^. In agreement with these findings, TSA decreased the PHA-induced proliferation without affecting Ca^2+^ signaling in T cells. Furthermore, the antiproliferative effect of delphinidin was abolished in presence of TSA. Altogether, our results suggest that modulating HDAC activity might be an additional mechanism by which delphinidin exerts its antiproliferative effect on T cells. In line with these results, delphinidin has been shown to modulate the balance between HDAC and histone acetyltransferase (HAT) by inhibiting HAT activity in MH7A cells^21^. Delphinidin ability to decrease [Ca^2+^]_i_ was not modified in presence of TSA suggesting that its effect on HDAC is downstream of [Ca^2+^]_i_ signaling.

The present study identified ERα as the key receptor transducing antiproliferative effect exerted by delphinidin on T lymphocytes. Indeed, we show that the antiproliferative properties of delphinidin, as well as its effects on Ca^2+^ signaling and ERK1/2 pathway, were abolished after pharmacological blockade of ER with fulvestrant. Most importantly, deletion of ERα completely abolished the effects of delphinidin on proliferation and Ca^2+^ signaling of murine PBMCs. These results are in agreement with previous study in which ERα has been identified to mediate delphinidin beneficial effects on endothelial cells^14^. We provide further evidence of ERα as a target of delphinidin on T lymphocytes.

Interestingly, delphinidin decreased the PHA-induced proliferation and the thapsigargin-induced [Ca^2+^]_i_ increase in T cells from patients with cardiovascular risk factors but not from MetS patients. A negative correlation was found between delphinidin capacity to decrease the PHA-induced proliferation and the number of MetS criteria. This differential effect might be explained by alterations of ERα expression on T lymphocytes from MetS patients compared to healthy subjects and non-MetS patients. Indeed, the expression of ERα is lower in T cells of patients with chronic inflammatory diseases like systemic lupus erythematosus, which is characterized by wide-spread inflammation with high pro-inflammatory cytokines^42, 43^. Similarly, ERα expression is decreased in splenocytes of animals subjected to trauma and hemorrhage, a general inflammatory condition, compared to control animals^44^. In addition, others studies have reported a decreased inflammation-associated ERα expression in other cell types^45–47^. These studies support a concept of down-regulation of ERα under inflammatory conditions, which might lead to a reduced signaling through ERα pathways.

In addition to T cell proliferation increase, diet-related metabolic disorders are associated with increased T lymphocyte differentiation toward pro-inflammatory subsets with relative decline in anti-inflammatory subsets^48^. Delphinidin decreased the differentiation of T cells from healthy subjects toward Th1, Th17, Treg but not Th2 subsets. Indeed, delphinidin treatment decreased the expression of Th1, Th17 and Treg subsets-specific transcription factors as well as the cytokines produced by these respective T subsets. Interestingly, delphinidin had no effect on Th2-specific transcription factor or Th2-signature cytokine. This might be due to the difference in IP_3_ and Ca^2+^ signaling in Th2 cells compared to other CD4^+^ subsets. Indeed, TCR activation is not able to increase IP_3_ and [Ca^2+^]_i_ in Th2 cells^49^. Furthermore, the inhibitory effects of delphinidin on Th17 and Treg differentiation were moreover conserved in T cells isolated from patients with cardiovascular risk factors. It is noteworthy that delphinidin effect on IL-17 production is negatively correlated with the number of MetS criteria.

Delphinidin was able to decrease the production of pro-inflammatory cytokine IL-2 in T cells from non-MetS and MetS patients. NFAT can act with multiple transcriptional partners (AP-1, NfK-B, STAT3, STAT5, Smad2/3) and targets (IL-2, T-bet, GATA3, R RORγt and FOXP3)^50^. In the present study, NFAT signaling participates on the effect of delphinidin. Indeed, in T cells from non-MetS patients, delphinidin inhibited not only IL-2 but also RORγt and FOXP3, all of which are targets of NFAT. Thus, delphinidin inhibited some, but not all, of NFAT target genes, such as T-bet and GATA3, in T cells from non-MetS patients. However, in T cells from MetS, delphinidin was not able to inhibit any of the targets tested including T-bet, GATA3, RORγt and FOXP3, except for IL-2. Thus, the mechanism by which delphinidin is still able to inhibit IL-2 secretion in T cells from MetS patients might be due to a mechanism independent to NFAT activation.

Despite the fact that the patients with one or two MetS criteria (non-MetS patients) cannot be classified as MetS patients, they can be classified as patients with cardiovascular diseases risk factors. Thus, delphinidin can decrease T cell proliferation and modulate T cell differentiation in healthy subjects and in patients with cardiovascular diseases risk factors.

Taken together, our results suggest that delphinidin might be an immuno-modulatory and anti-inflammatory agent that can alter T lymphocyte proliferation and differentiation. The present study identifies ERα as one of key receptor transducing immuno-modulatory effect exerted by delphinidin. In addition to its preventive immunomodulatory effects in healthy subjects, delphinidin may have beneficial effects on processes leading to chronic inflammation associated with T lymphocyte activation in patients with cardiovascular risk factors. Immuno-modulation of the immune response by delphinidin can provide an alternative or complementary approach in the treatment of chronic inflammatory metabolic disorders caused by inappropriate or excessive T lymphocyte responses.

## Methods

### Reagents

Delphinidin chloride was purchased from Extrasynthèse (Genay, France) and was used at 10^−2^ g/L. This concentration has been described to induce the maximal relaxing effect on *ex vivo* rat aorta^13^, to prevent angiogenesis through an inhibition of migration and proliferation^16, 17^ and to inhibit endothelial apoptosis^51^. Delphinidin was diluted in dimethylsulfoxide (DMSO) from Sigma Aldrich (St Louis, MO). The final concentration of DMSO in experiments never exceeded 0.1%. Anti-CD3 (clone OKT3) and anti-CD28 (clone CD28.2) human antibodies were purchased from BioLegend® (San Diego, CA). Histopaque®1077, Histopaque®1083, thapsigargin, Phytohemagglutinin (PHA), phorbol-12-myristate-13-acetate (PMA), ionomycin, fulvestrant, and SKF96365 were purchased from Sigma-Aldrich. Mibefradil hydrochloride was purchased from Abcam (Cambridge, UK) and trichostatin A (TSA) from Santa Cruz Biotechnology (Santa Cruz, CA). U0126 was obtained from Calbiochem (San Diego, CA). RPMI-1640, Na-pyruvate, non-essential amino-acid (NEAA) and penicillin/streptomycin were purchased from Lonza (Basel, Switzerland). Fetal bovine serum (FBS) and Fluo-4 acetoxymethyl ester (AM) were purchased from Life Technologies (Carlsbad, CA).

### Human subjects

Human T lymphocytes were isolated from buffy coat from healthy donors obtained through the Etablissement Français du Sang (EFS Pays de la Loire, Nantes, France). Blood samples were collected and processed following standard ethical procedures after obtaining written informed consent from each donor under EFS contract N° ANG 2013–03 and approval for this study by the Ethics Committee of the University Hospital of Angers (France). MetS patients were eligible for inclusion, according to the European definition of the International Diabetes Federation (IDF)^52^, when they had at least three criteria out of the five following: (i) a waist circumference ≥94 or 80 cm for men and women, respectively; (ii) high systolic and diastolic pressures ≥130/85 mm Hg or antihypertensive treatment; (iii) fasting glycemia ≥1.0 g/L or anti-diabetic treatment; (iv) triglycerides ≥1.5 g/L and (v) high-density lipoprotein (HDL) <0.4 g/L in men or <0.5 g/L in women or cholesterol-lowering drug treatment. Patients were classed on two groups depending of the number of MetS criteria that they displayed: (i) patients with one or two criteria (non-MetS patients), and (ii) patients with more than 3 criteria (MetS patients). Non-MetS and MetS patients from the METABOL cohort were included at the Department of Endocrinology and Nutrition of the University Hospital of Angers (NCT 00997165). See Table 1 for clinical parameters.

**Table 1:** Baseline characteristics of non-MetS (n = 24) and MetS patients (n = 46).

	Non-MetS patients	MetS patients
Number	24	46
Mean age (years)	54.9 ± 2.3	53.2 ± 1.6
Sex ratio (M/F)	11/13	24/22
BMI (kg/m^2^)	30.4 ± 1.1	31.5 ± 0.8
Waist size (cm)	101.06 ± 2.52	109.7 ± 2.8*
Waist/Hips ratio	0.95 ± 0.01	1 ± 0.02*
Systolic blood pressure (mm Hg)	124.2 ± 1.8	126.4 ± 1.9
Diastolic blood pressure (mm Hg)	77.9 ± 1.6	77.2 ± 1.5
Glycemia (g/l)	1.01 ± 0.02	1.2 ± 0.04**
HBA1c (%)	5.8 ± 0.05	6.4 ± 0.15***
Total cholesterol (g/l)	2.15 ± 0.09	1.7 ± 0.08
HDL cholesterol (g/l)	0.5 ± 0.02	0.42 ± 0.004**
LDL cholesterol (g/l)	1.29 ± 0.08	1.18 ± 0.018
Triglycerides (g/l)	1.08 ± 0.07	1.6 ± 0.2*
Number of MetS components (%)		
		—
1	38	—
2	62	—
3	—	41
4	—	50
5	—	9
Treatment (%)		
Oral antidiabetic	17	52
Antihypertensive	33	58
Statins	25	40

All patients were fasted before blood collection. All values are expressed in International System (SI) units. *P < 0.05, **P < 0.01 and ***P < 0.001.

### Mice

Animal study was carried out using approved institutional protocols (CEEA.2011.40) and was conformed the Guide for the Care and Use of Laboratory Animals published by U.S. National Institutes of Health (NIH Publication No. 85–23, revised 1996). C57BL/6 females ERα Wild Type (WT) or Knock-Out (KO) mice were used. They were ovariectomized at the age of 12 weeks (n = 4 animals for each group). After 7 days, blood sample was obtained from each animal by cardiac puncture following isoflurane anesthesia.

### PBMC isolation and cell culture

Peripheral blood mononuclear cells (PBMCs) were isolated from blood of human subjects or mice using Histopaque® 1077 or Histopaque® 1083, respectively. Diluted blood was carefully layered onto the Histopaque and centrifuged at 400 *g* for 30 min. After centrifugation, the opaque interface containing mononuclear cells was collected and washed three times with PBS (pH 7.2). Cells were then resuspended in RPMI 1640 medium with 10% heat-inactivated FBS. The adherent cells were depleted by incubation in culture dish for 2 h at 37 °C in 5% CO_2_ atmosphere. After isolation, PBMCs were resuspended at 10^6^ cells/mL in complete medium consisting of RPMI-1640 with ultraglutamine1 supplemented with, 1% penicillin/streptomycin, 1% NEAA, 1% Na-pyruvate and 10% FBS.

### Cell phenotyping

Freshly isolated PBMCs were adjusted to a density of 1 × 10^6^ cells/mL and labeled for 20 min at room temperature with PE- or FITC- conjugated monoclonal antibodies against CD45, CD3, CD4 and CD8 antigens (markers for leukocytes, T lymphocytes, CD4^+^ and CD8^+^ helper T cells, respectively) (Beckman Coulter, Villepinte, France). The cells were washed and resuspended in PBS, then 10,000 events were analyzed in a flow cytometer 500 MPL system and results were analyzed with CXP software (Beckman Coulter). 93.5 ± 3.7% of isolated cells were leukocytes. The purity of CD3^+^ human T lymphocytes was 77.5 ± 7.4%, CD4^+^ and CD8^+^ T lymphocytes represented 59.7 ± 5.9% and 23.2 ± 2.4%, respectively, of total isolated cells (Supplementary Fig. 6). Since isolated PBMCs are enriched in T cells, the term T lymphocytes will be used to describe isolated PBMCs.

### Cell proliferation assay

T lymphocytes were cultured in triplicates in 96-well culture plates at 10^4^ cells/well in a total volume of 0.2 mL. Cells were treated with DMSO (final concentration 0.1%, used as control), anti-CD3 plus anti-CD28 antibodies (10 µg/mL and 5 µg/mL, respectively) in absence or in presence of delphinidin (10^2−^g/L) and incubated in 5% CO_2_-air humidified atmosphere at 37 °C for 24 and 48 h. Indeed, we have performed a proliferation test using PHA for 24, 48 and 72 h to choose the most appropriate incubation time. Our results showed that, when stimulated with PHA, the number of T cells proliferating peaked at 48 h, under the experimental condition used. The ability of PHA to induce T cell proliferation after 72 h of treatment and that of delphinidin to abrogate the response were identical than those obtained at 48 h (data not shown). Proliferation rate was assessed by using CyQUANT® NF Cell Proliferation Assay Kit (Life Technologies, Carlsbad, CA) according to the manufacturer’s instruction. Stimulation was also induced in the presence of PHA (5 µg/mL) or PMA (10 ng/mL) plus ionomycin (1 µM). To assess the potential implication of SOCE, HDAC and ER in delphinidin effects, T cells were pre-incubated for 30 min prior to stimulation by SOCE inhibitor (SKF96365, 10 µM), HDAC inhibitor (Trichostatin A, 100 nM) or nonselective ER antagonist (fulvestrant, 100 nM), respectively. The inhibition of proliferation by delphinidin was calculated by determining the decrease in cell proliferation when T cells were treated with PHA and delphinidin compared to PHA alone.

### Cell cycle analysis

T lymphocytes were cultured in 12-well culture plates at 10^6^ cells/mL in a total volume of 2 mL. Then, they were treated with PHA (5 µg/mL) in absence or in presence of delphinidin (10^−2^ g/L) for 48 h. After treatment, cells were washed twice in PBS and 0.2 mL of nuclear isolation medium (50 µg/mL propidium iodide, 0.6% NP-40, 100 µg/mL RNase, in PBS) was added. Cells were then incubated (1 h) at room temperature in the dark before addition of 0.2 mL PBS and analyzed by flow cytometry. The distribution of cells in G_0_/G_1_, S and G_2_/M phases was determined using CXP analysis software (Beckman Coulter).

### Measurement of [Ca^2+^]_i_

Isolated T lymphocytes were loaded with fluorescent Ca^2+^ indicator dye Fluo-4 AM (3 µM) (40 min at 37 °C in the dark in complete culture medium). The cells were then washed twice and suspended in physiological salt solution (PSS), pH 7.4, (composition in mM: NaCl 119, KCl 4.75, CaCl_2_ 1.25, MgSO_4_ 1.17, KH_2_PO_4_ 1.18, NaHCO_3_ 25, glucose 11). The cells were then transferred to opaque 96-well culture plate and stimulated with thapsigargin (1 µM), delphinidin (10^−2^ g/L), or both. [Ca^2+^]_i_ was determined by fluorometric reading during 20 min performed with Mithras LB 940 Multimode Microplate Reader (Berthold Technologies), and the method of Grynkiewicz *et al*.^53^ was used to calibrate the *in vitro* [Ca^2+^]_i_ as previously described^15^. For Ca^2+^-free experiments, CaCl_2_ was omitted from PSS and 0.5 mM EGTA was added 10 min before measurement. To assess Ca^2+^ release from intracellular stores, cells were stimulated, as mentioned above, in free-Ca^2+^ PSS and the Ca^2+^ release from intracellular stores was followed for 20 min. Thereafter, CaCl_2_ (1.25 mM) was added to obtain an influx of extracellular Ca^2+^. The potential implication of SOCE, HDAC and ER in delphinidin effects was tested by incubating cells for 10 min prior to stimulation by SKF96365 (10 µM), Trichostatin A (100 nM) or Fulvestrant (100 nM), respectively, then the previous experimental protocol was repeated. Also, the implication of voltage-operated Ca^2+^ channels was studied by using mibefradil (3 µM) at maximally active concentration.

### Western blot

After treatment, T lymphocytes were harvested, washed twice in PBS and lysed with ice-cold RIPA buffer containing protease inhibitor on ice for 15 min. The lysates were centrifuged at 15,000 *g* for 15 min at 4 °C. The supernatants were collected and quantified for proteins concentration. Twenty-five µg proteins were separated on NuPAGE™ 4–12% Bis-Tris gels (Life Technologies, Carlsbad, CA). The separated proteins were transferred onto nitrocellulose membranes (GE Healthcare, Pittsburg, PA) and blotted according to standard procedures. Blots were probed with phosphorylated or total-ERK1/2 antibodies (Cell Signaling Technology). Monoclonal anti-β-actin antibody (Sigma-Aldrich) was used to visualize protein gel loading. Membranes were then incubated with the appropriate horseradish peroxidase-conjugated secondary antibodies. Protein-antibody complexes were detected by enhanced chemiluminescence method using SuperSignal™ West Femto Maximum Sensitivity Substrate (Thermo Scientific) with a Chemi-Smart 5000 imager system (Vilber-Lourmat, Marne-la-Vallée, France). Blots were quantified by densitometry using ImageJ software.

### NFAT activation assay

T lymphocytes treated with appropriate stimulus were harvested, washed twice in PBS and nuclear extracts were prepared as previously described^54^. Cells were resuspended in 200 µL of Buffer A containing 10 mM HEPES (pH 7.9), 1.5 mM MgCl_2_, 10 mM KCl, with protease and phosphatase inhibitors mixture. Cells were lysed on ice for 15 min and centrifuged at 1,000 *g* for 10 min at 4 °C. The supernatant was saved as cytosolic fraction. The nuclear pellet was resuspended into 100 µL of Buffer B containing 20 mM HEPES (pH 7.9), 25% glycerol, 420 mM NaCl, 1.5 mM MgCl_2_, 0.2 mM EDTA with protease and phosphatase inhibitors mixture. Then, samples were incubated on ice for 30 min followed by brief sonication and centrifugation at 15,000 *g* for 30 min at 4 °C. The supernatant containing the nuclear extracts was collected and the protein concentration was quantified. Fifteen µg of nuclear extracts were tested for the NFAT activation using TransAM^**®**^ NFATc1 transcription factor kit (Active Motif, Carlsbad, CA) according to the manufacturer’s instruction.

### Intracellular transcription factors and cytokines staining

Staining of transcription factors was performed on T cells stimulated for 24 h with delphinidin (10^−2^ g/L), PHA (5 µg/mL) or both. Cells were fixed and permeabilized using fixation and permeabilization buffers, respectively (BioLegend) according to the manufacturer’s instructions, then they were stained for 45 min with FITC-conjugated anti-T-bet Ab, PE-conjugated anti-GATA3 Ab, FITC-conjugated anti-FOXP3 Ab (BioLegend) and PE-conjugated anti-RORγt Ab (Milteny BiotecGmbH, Bergisch Gladbach, Germany). The cells were then analyzed by flow cytometry (Beckman Coulter) and data were analyzed with Flowing Software 2.

Alternatively, intracellular production of IFNγ, IL-4, IL-17A and IL-10 (produced by Th1, Th2, Th17 and Treg, respectively), in addition to IL-2 was analyzed in T cells. For this purpose, T cells were stimulated for 5 h with delphinidin (10^−2^ g/L), PMA (50 ng/mL) plus ionomycin (1 µg/mL), or both, in the presence of 5 µg/mL brefeldin A (BFA) (BioLegend) for the final 3 h of culture. BFA leads to the accumulation of most cytokines inside the cell by blocking transport processes during cell activation^55^. FITC-conjugated anti-human IL-2 Ab, PE/Cy7-conjugated anti-human IFNγ Ab, APC-conjugated anti-human IL-4 Ab, FITC-conjugated anti-human IL-17A Ab and APC-conjugated anti-human IL-10 Ab (BioLegend) were used to stain cytokines. Cells were then washed and data were acquired using MACSQuant Analyzer and analyzed with MACSQuantify™ Software (Miltenyi Biotec).

### RNA isolation and analysis of transcription factors expression

T lymphocytes isolated from non-MetS and MetS patients were treated with DMSO (used as control), PHA (5 µg/mL) in absence or in presence of delphinidin (10^−2^ g/L) and incubated in 5% CO_2_-air humidified atmosphere at 37 °C for 24 h. Cells were collected and stored at −80  °C until analysis. Total cellular RNA was extracted and purified using RNeasy Micro Kit (QIAGEN) then treated with RNase-free DNase I (QIAGEN) and reverse transcribed with SuperScript^TM^ II Reverse Transcriptase (Invitrogen) according to the manufacturer’s instructions. Quantitative analysis of expression of T-bet, GATA3, RORγt and FOXP3 genes was performed using LightCycler^®^ 480 System (Roche) in 384-well optical plates with Maxima SYBR Green qPCR Master Mix (Thermofisher Scientific) according to the manufacturer’s instructions. The PCR primers were provided by Eurofins MWG. Samples were analyzed in duplicate and relative expression levels of target genes were normalized to expression of RPL13a.

### Cytokines measurement

T lymphocytes isolated from non-MetS and MetS patients were treated with DMSO (used as control), PHA (5 µg/mL) in absence or in presence of delphinidin (10^−2^ g/L) and incubated in 5% CO_2_-air humidified atmosphere at 37 °C for 24 h. Supernatants were collected and stored at −80 °C until analysis. Levels of IL-2, IFNγ, IL-4, IL-17A, IL-10 and IL-6 in supernatants were analyzed using Human Magnetic Luminex® Screening Assay (R&D Systems, France) on Bio-Plex 200 System (Bio-Rad). Manufacturer-specified limits of detection were 2.23, 1.27, 4.46, 1.10, 0.30 and 1.11 pg/mL for IL-2, IFNγ, IL-4, IL-17A, IL-10 and IL-6, respectively. The percent inhibition of IL-17 production by delphinidin was calculated by determining the decrease in IL-17 production when T cells were treated with PHA and delphinidin compared to PHA alone.

### Statistical analysis

Data were expressed as means ± SEM. *n* refers to the number of experiments. Statistical analyses were performed with Graph-Pad Prism Software Version 5.0 using non-parametric Mann-Whitney test. p < 0.05 was considered statistically significant.

## Electronic supplementary material

Supplementary Information
