# Supplementary material for: Estrogen receptor α/HDAC/NFAT axis for delphinidin effects on proliferation and differentiation of T lymphocytes from patients with cardiovascular risks
Source: Sci Rep. 2017 Aug 24;7:9378. doi: 10.1038/s41598-017-09933-4 (PMC5570903; doi:10.1038/s41598-017-09933-4)
Supplement: Supplementary file 1 — Supplementary Information [file 41598_2017_9933_MOESM1_ESM.pdf]

Estrogen receptor α/HDAC/NFAT axis for delphinidin effects on proliferation and differentiation of T lymphocytes from patients with cardiovascular risks

Ousama Dayoub, Soazig Le Lay, Raffaella Soleti, Nicolas Clere, Gregory Hilairet, Séverine Dubois, Frédéric Gagnadoux, Jérôme Boursier, Maria Carmen Martínez, Ramaroson Andriantsitohaina

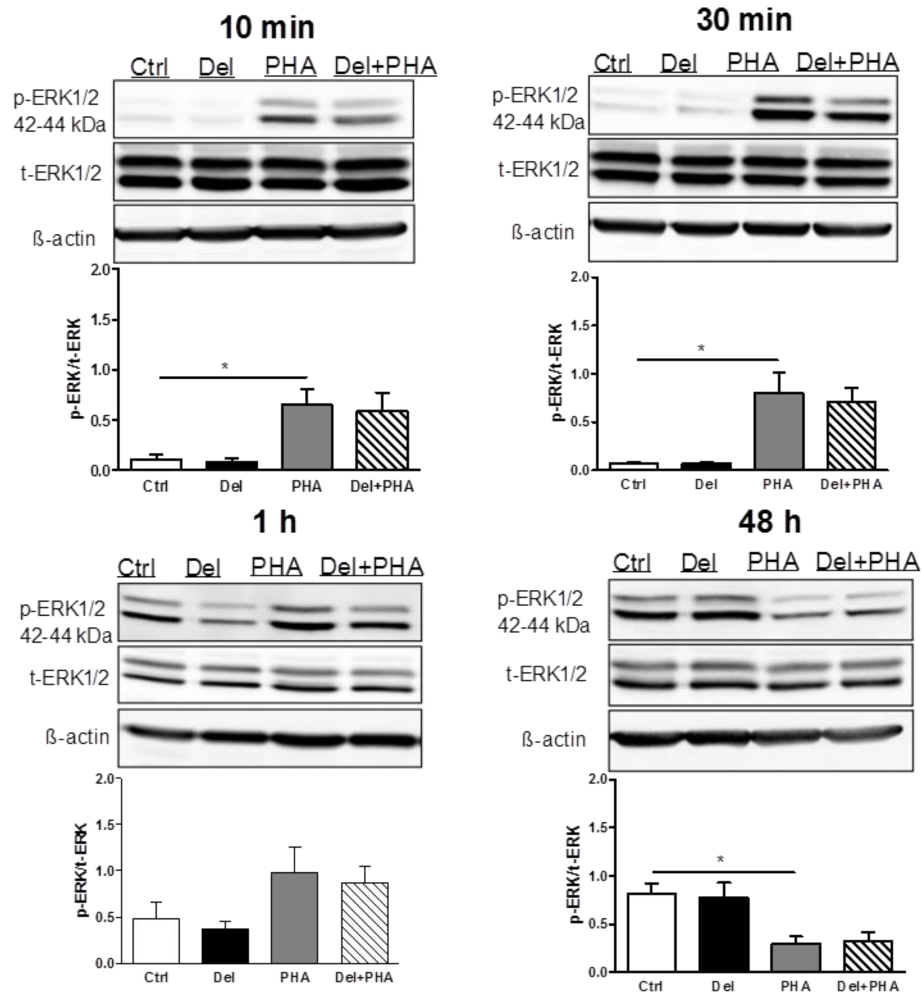

Supplementary Figure 1. Effects of delphinidin on ERK1/2 pathway activation of T lymphocytes from healthy subjects. Western blot of phosphorylated ERK1/2 (p-ERK1/2) in T cells exposed to either 10^{-2} g/L of delphinidin (Del), 5µg/mL PHA or both during indicated time. Histograms show densitometric analysis of phosphorylated ERK1/2 expression normalized to total ERK1/2 (t-ERK1/2) expression. Data represent the mean ± SEM (*n*=4-8). **P*<0.05.

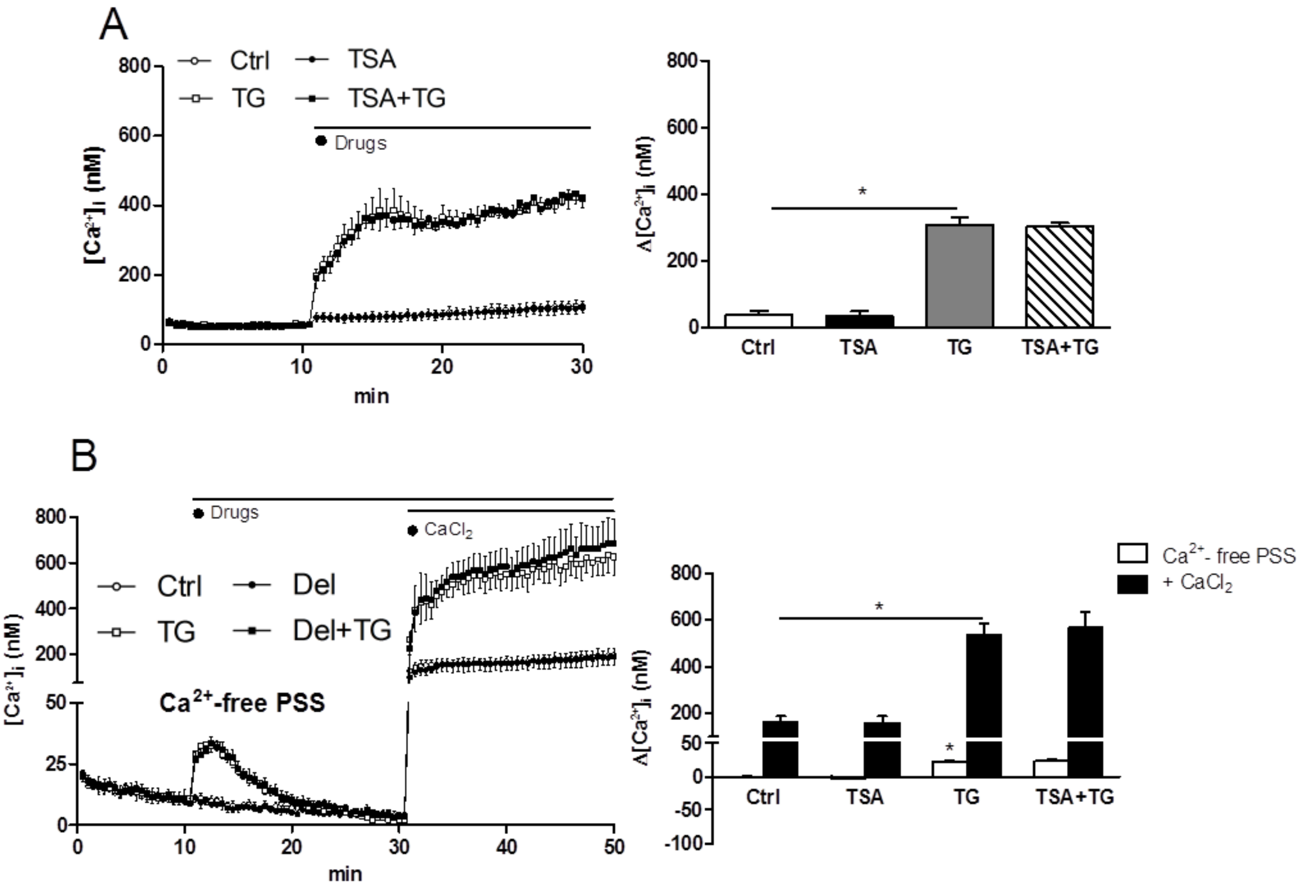

Supplementary Figure 2. Effect of HDAC inhibitor on $[Ca^{2+}]_i$ of T lymphocytes from healthy subjects. (A) Representative traces (*left*) showing the effect of 100 nM of trichostatin A (TSA) alone or after activation by 1μM thapsigargin (TG) on $[Ca^{2+}]_i$ in Ca^{2+}-containing PSS, histogram (*right*) showing the mean of the responses induced by 100 nM of TSA alone or after activation by TG in Ca^{2+}-containing PSS. **(B)** Representative traces (*left*) showing the effect of TSA on $[Ca^{2+}]_i$ increase induced by 1.25 mM of $CaCl_2$ after depletion of intracellular stores in Ca^{2+}-free PSS by TG, and histogram (*right*) showing the mean of the responses induced by TSA alone or in combination with TG in Ca^{2+}-free PSS and the subsequent addition of $CaCl_2$. Data are the mean ± SEM (*n*=4). *P<0.05 versus control group.

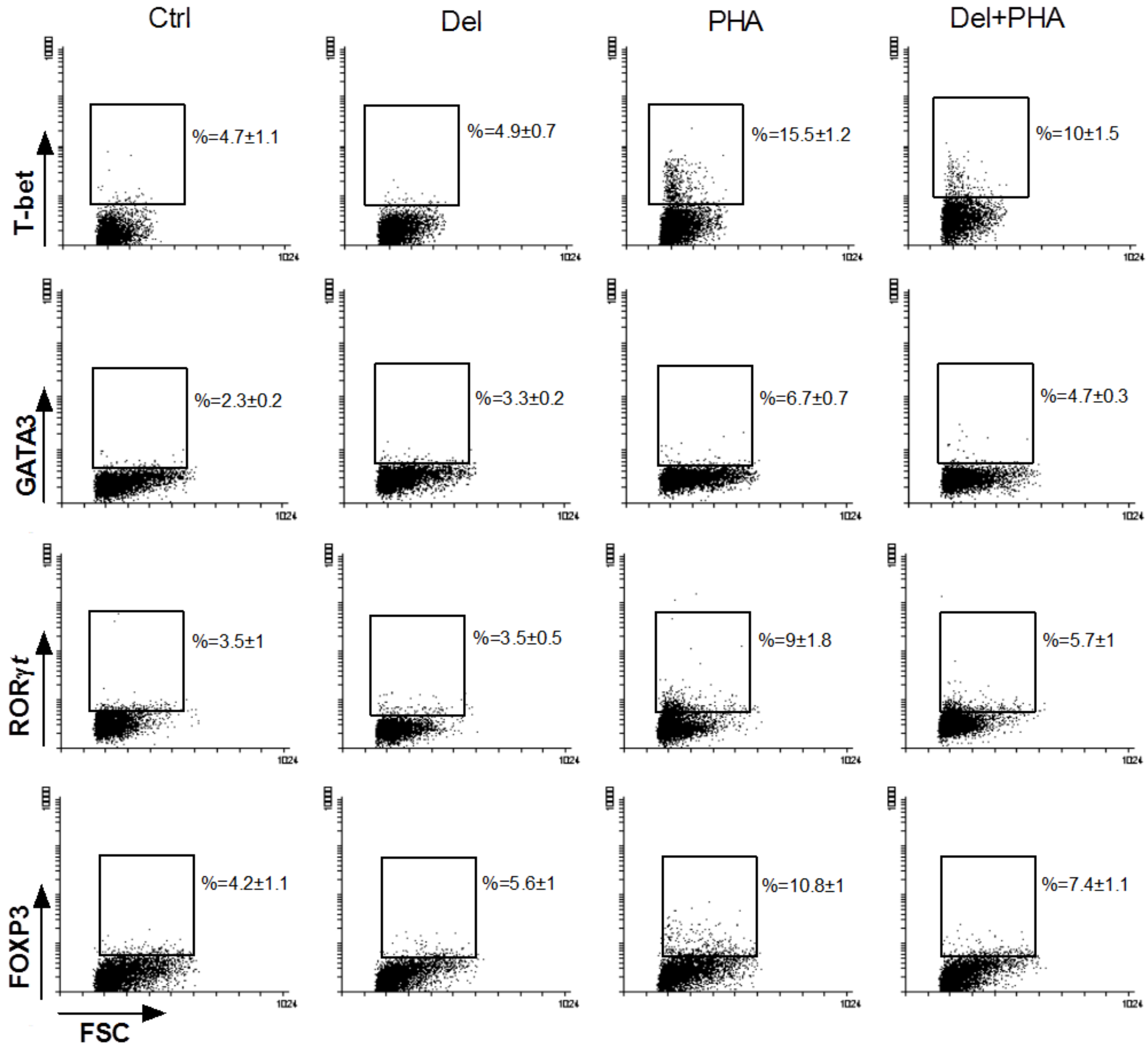

Supplementary Figure 3. Effect of delphinidin on transcription factors expression of T lymphocytes from healthy subjects. T cells were stimulated for 24 h with 10^{-2} g/L of delphinidin (Del), 5 µg/mL of PHA or both and stained for T-bet, GATA3, RORγt and FOXP3 transcription factors. Representative dot plots showing the percentage of positive cells for T-bet, GATA3, RORγt and FOXP3.

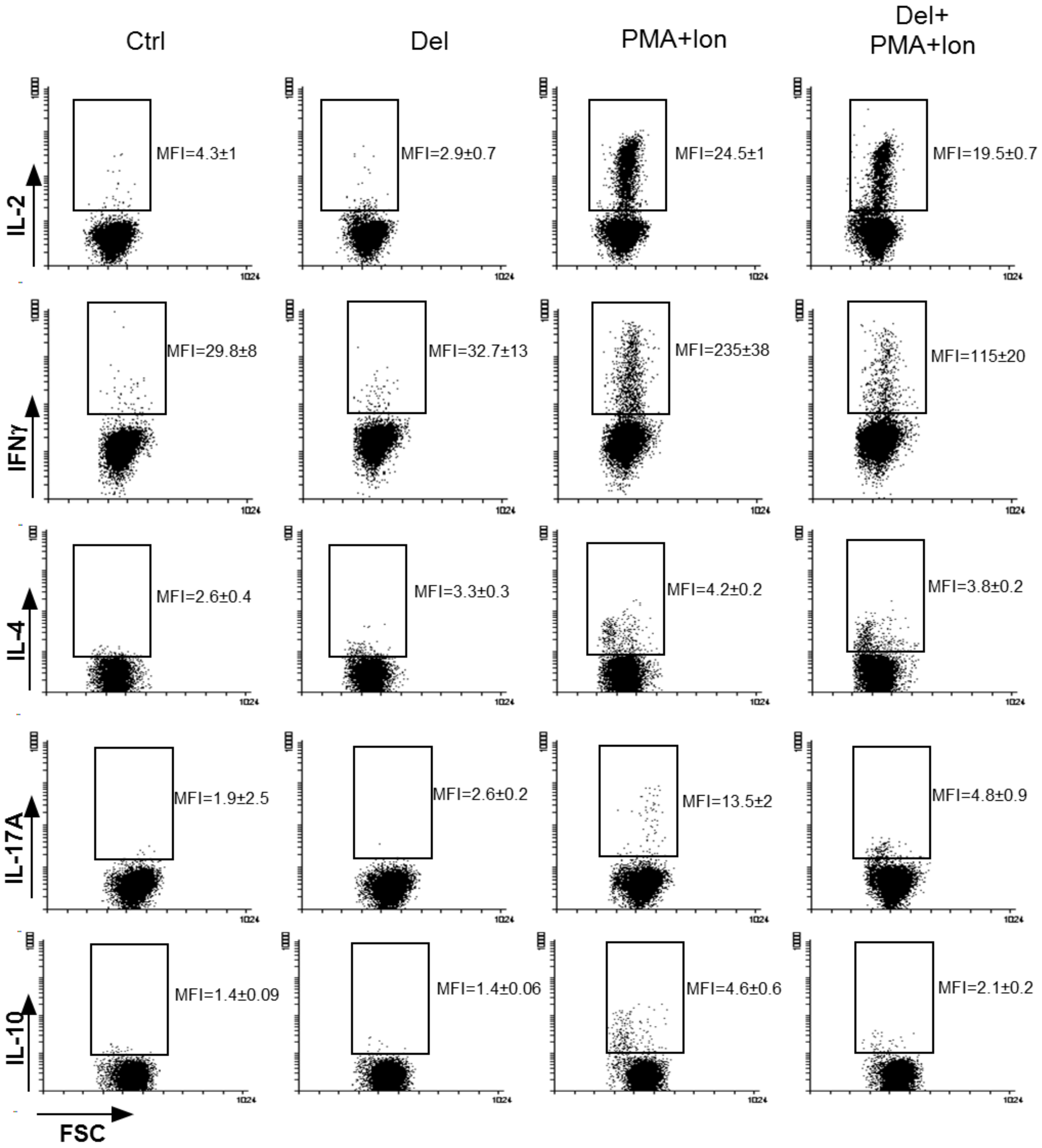

Supplementary Figure 4. Effect of delphinidin on cytokines production of T lymphocytes from healthy subjects. T cells were stimulated for 5h with 10^{-2} g/L of delphinidin (Del), 50 ng/mL phorbol-12-myristate-13-acetate (PMA) plus 1µg/mL ionomycin (Ion) or both, in the presence of 5µg/mL brefeldin A for the final 3h of culture and stained for IL-2, IFNγ, IL-4, IL-17A and IL-10 cytokines. Representative dot plots showing the fluorescence intensity of positive cells for IL-2, IFNγ, IL-17A, IL-4 and IL-10, respectively.

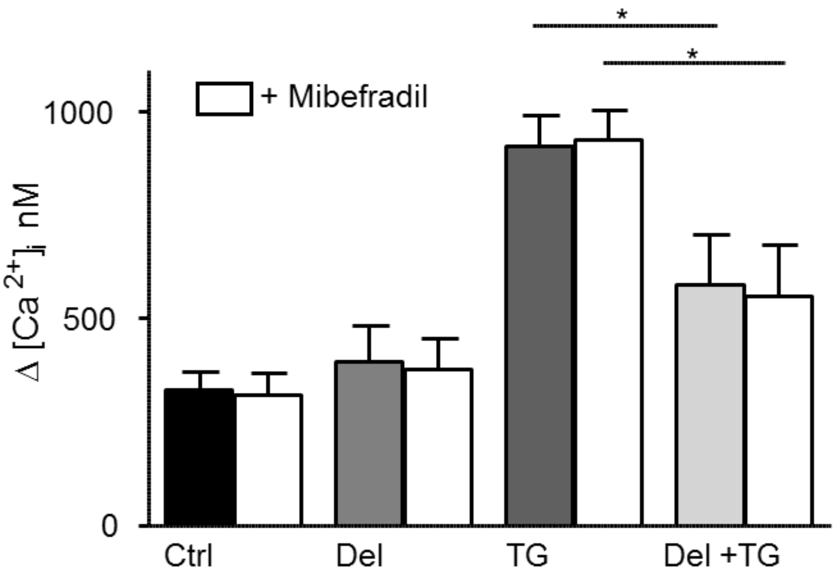

Supplementary Figure 5. Effects of mibefadril (3 µM) in the Ca^{2+} response induced by delphinidin (Del). Histograms showing the mean of the responses induced by the effect of 10^{-2} g/L Del alone or after activation by 1 µM thapsigargin (TG) on $[Ca^{2+}]_i$ increase in Ca^{2+}-containing PSS of T cells isolated from healthy subjects. *n*=3 in triplicate. **P*<0.05

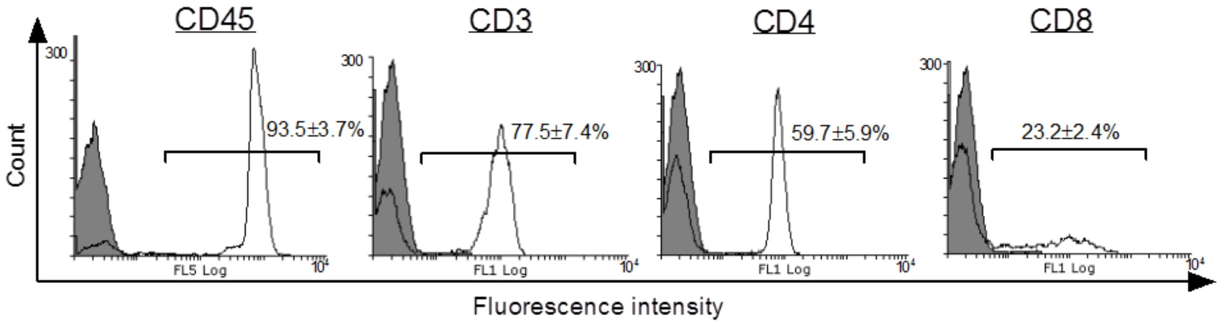

Supplementary Figure 6. Peripheral blood mononuclear cells phenotyping by flow cytometry. Representative flow cytometry data showing the percentage of cells expressing CD45, CD3, CD4 and CD8 in total cells isolated from healthy subjects. Cells were gated based on forward and side scatter followed by specific staining with the indicated antibodies. Gray histograms represent the negative controls lacking the indicated antibody, *n*=6.

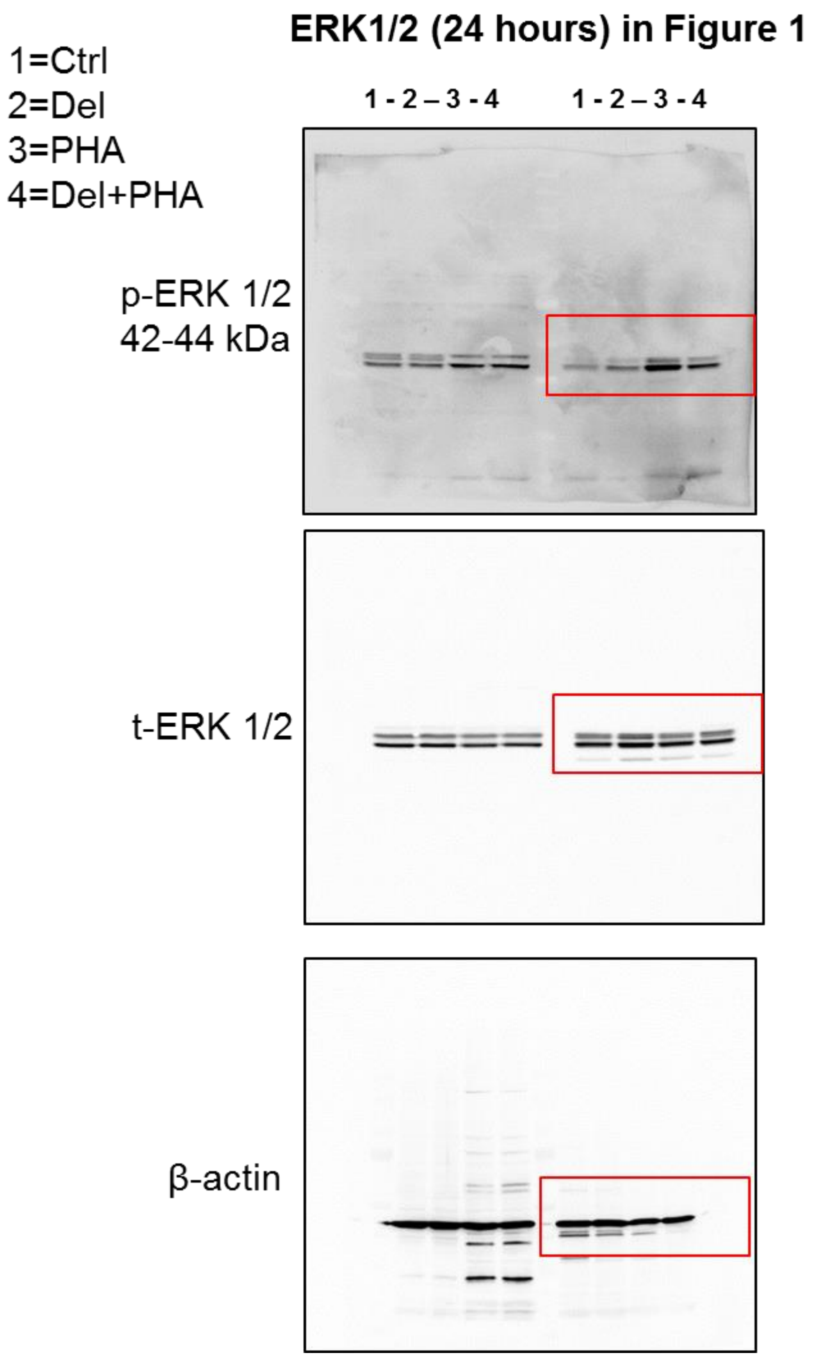

Supplementary Figure 7. Unprocessed images of the key immunoblots of Figure 1. Boxes indicate image areas shown in the indicated panels.

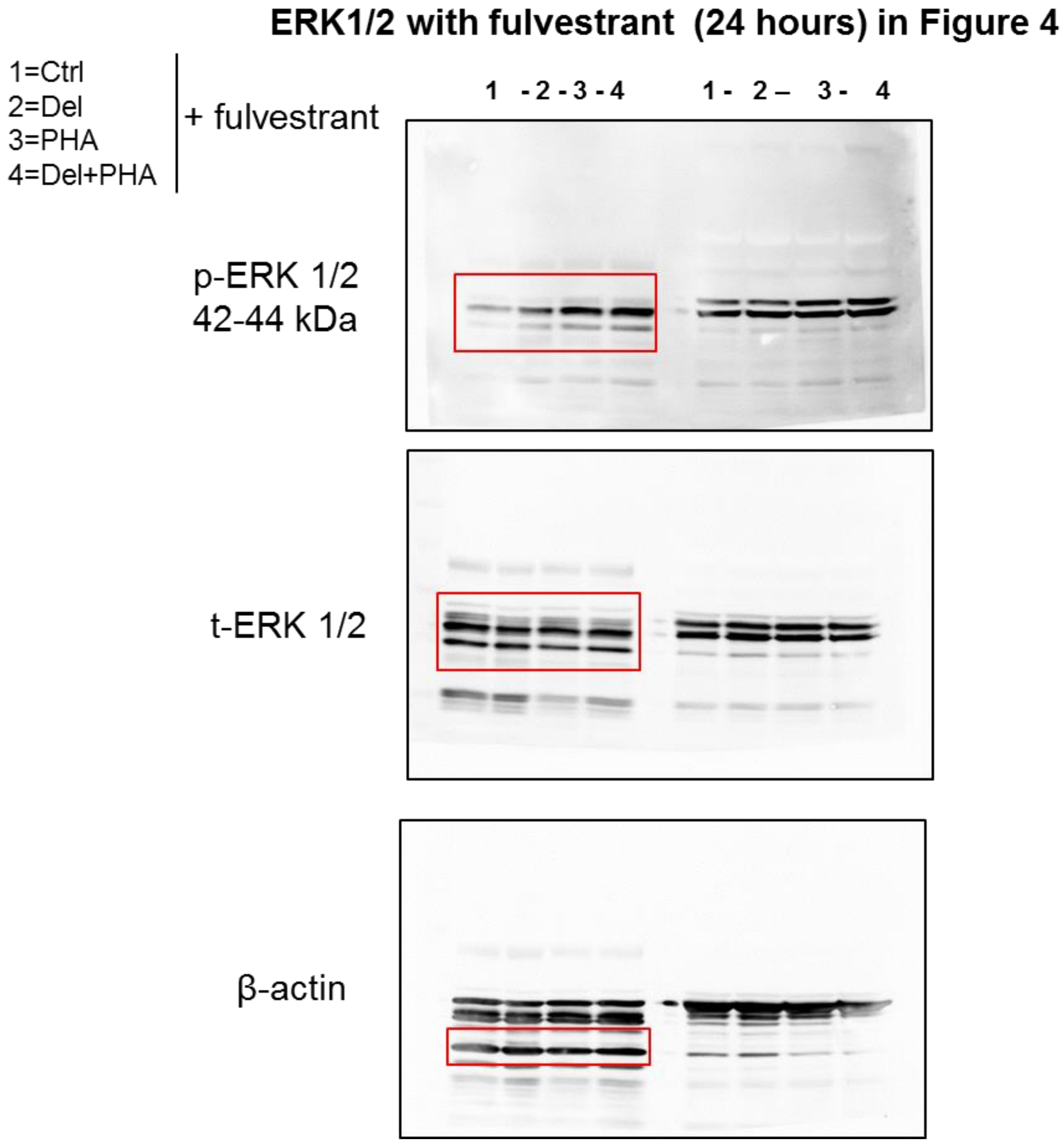

Supplementary Figure 8. Unprocessed images of the key immunoblots of Figure 4.

Boxes indicate image areas shown in the indicated panels.

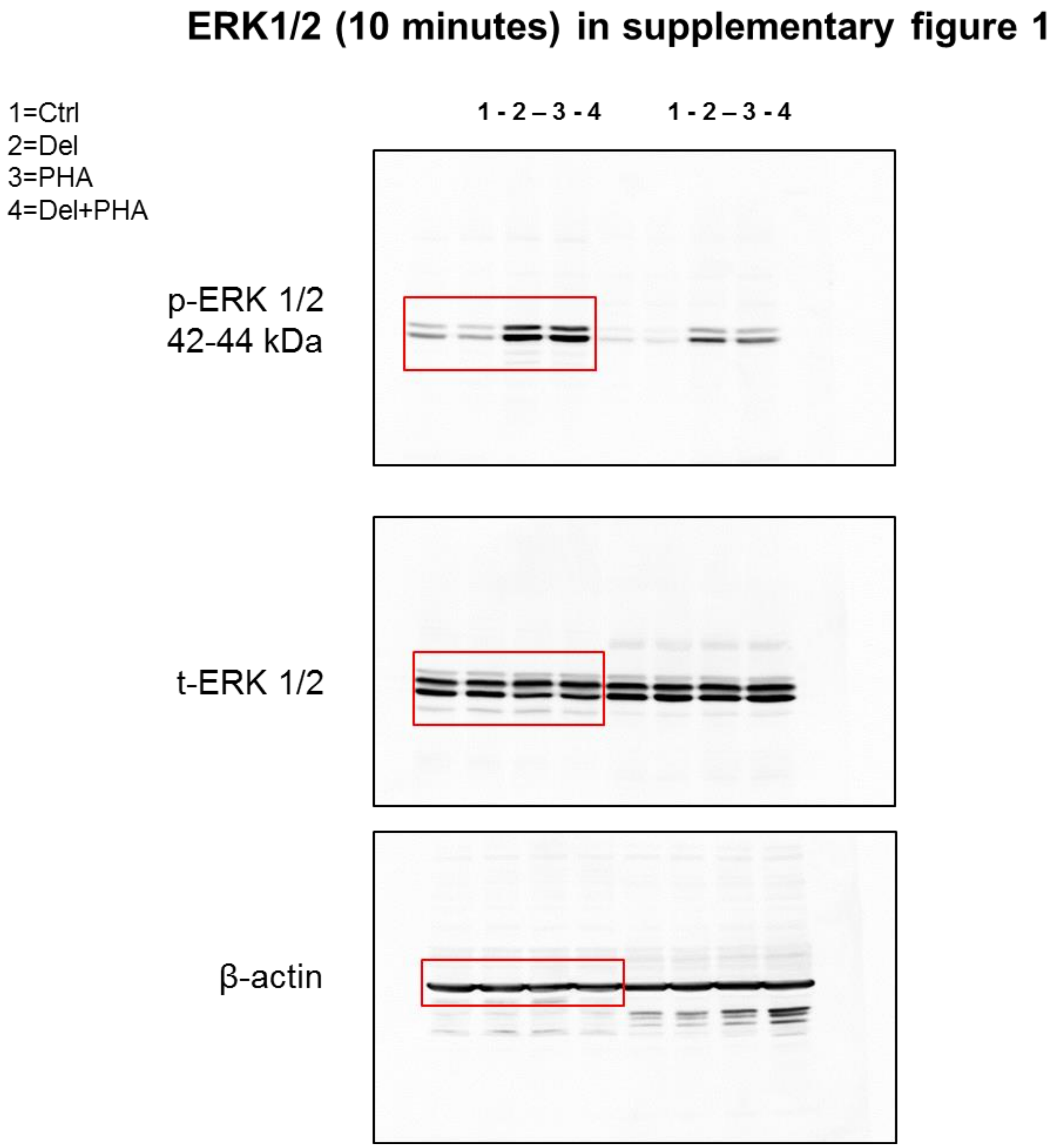

Supplementary Figure 9. Unprocessed images of the key immunoblots of Supplementary Figure 1. Boxes indicate image areas shown in the indicated panels.

ERK1/2 (30 minutes) in supplementary figure 1

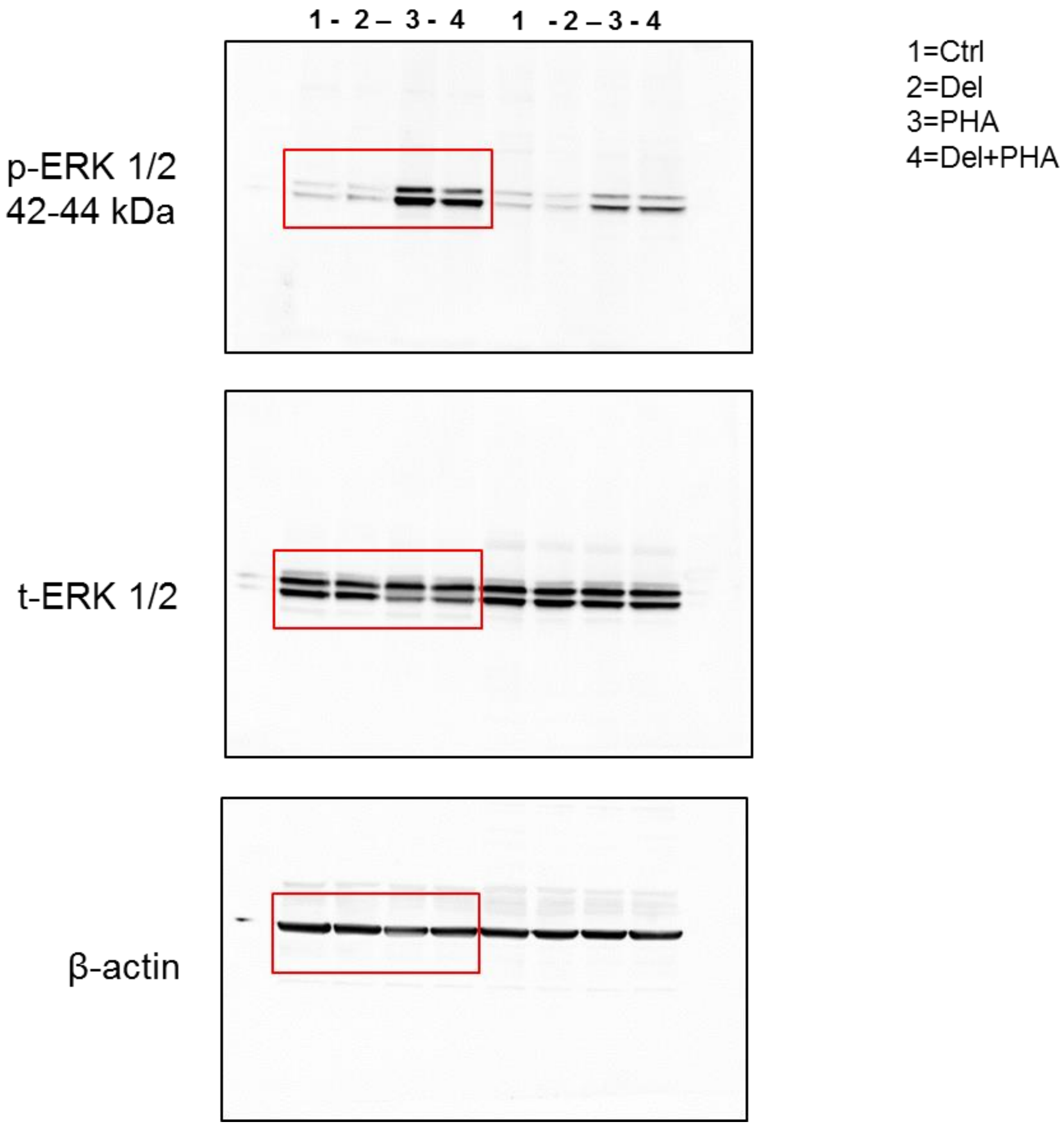

Supplementary Figure 10. Unprocessed images of the key immunoblots of Supplementary Figure 1. Boxes indicate image areas shown in the indicated panels.

ERK1/2 (1 hour) in supplementary figure 1

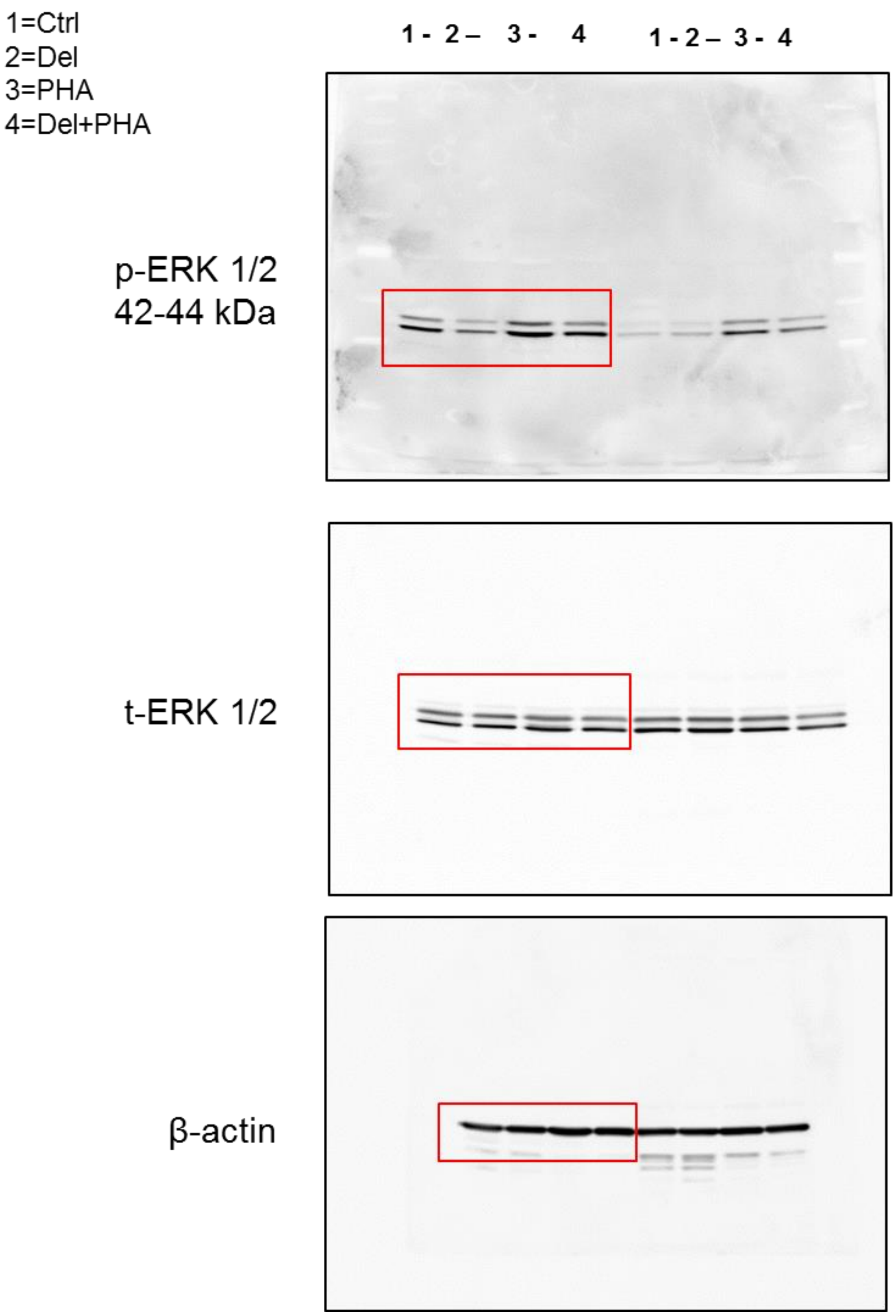

Supplementary Figure 11. Unprocessed images of the key immunoblots of Supplementary Figure 1. Boxes indicate image areas shown in the indicated panels.

ERK1/2 (48 hours) in supplementary figure 1

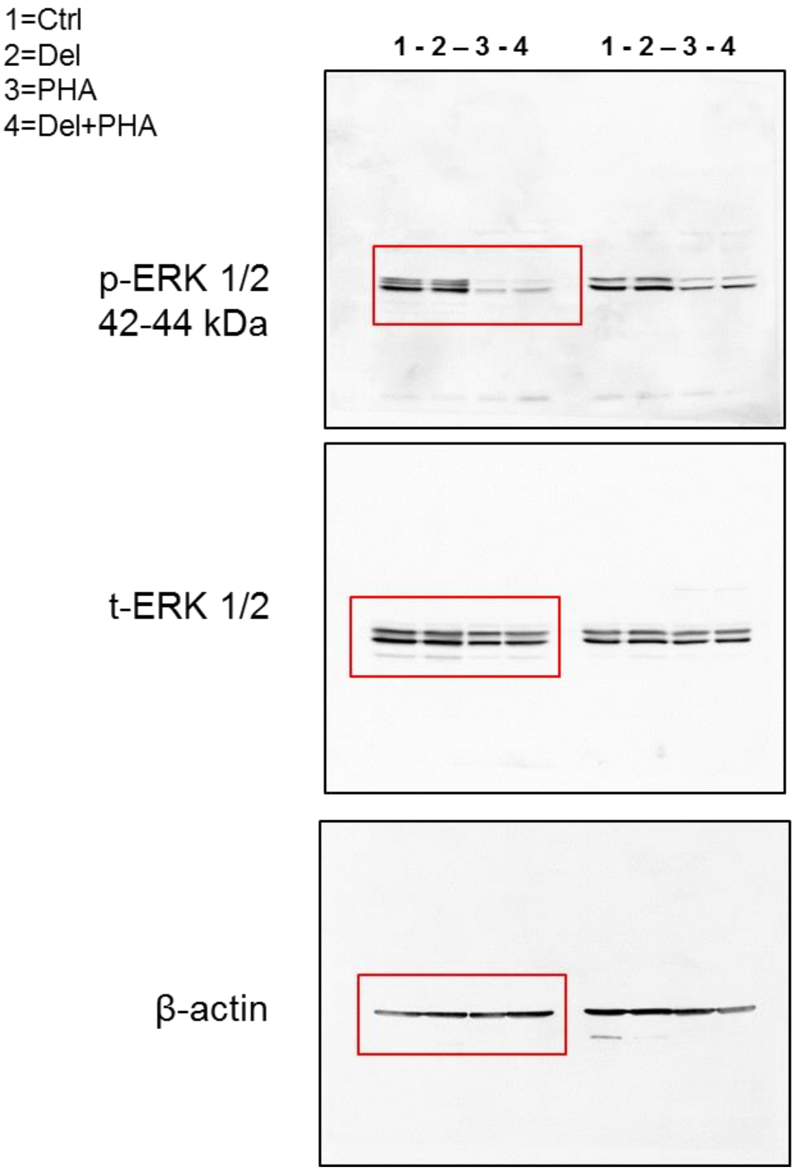

Supplementary Figure 12. Unprocessed images of the key immunoblots of Supplementary Figure 1. Boxes indicate image areas shown in the indicated panels.
